# Simultaneous Brain–Cervical Cord fMRI Reveals Intrinsic Spinal Cord Plasticity during Motor Sequence Learning

**DOI:** 10.1371/journal.pbio.1002186

**Published:** 2015-06-30

**Authors:** Shahabeddin Vahdat, Ovidiu Lungu, Julien Cohen-Adad, Veronique Marchand-Pauvert, Habib Benali, Julien Doyon

**Affiliations:** 1 Functional Neuroimaging Unit, University of Montreal, Montreal, Quebec, Canada; 2 SensoriMotor Rehabilitation Research Team (CIHR), Montreal, Canada; 3 École Polytechnique de Montréal, Montreal, Quebec, Canada; 4 INSERM/UPMC, Pitié-Salpêtrière Hospital, Paris, France; NIH NINDS, UNITED STATES

## Abstract

The spinal cord participates in the execution of skilled movements by translating high-level cerebral motor representations into musculotopic commands. Yet, the extent to which motor skill acquisition relies on intrinsic spinal cord processes remains unknown. To date, attempts to address this question were limited by difficulties in separating spinal local effects from supraspinal influences through traditional electrophysiological and neuroimaging methods. Here, for the first time, we provide evidence for local learning-induced plasticity in intact human spinal cord through simultaneous functional magnetic resonance imaging of the brain and spinal cord during motor sequence learning. Specifically, we show learning-related modulation of activity in the C6–C8 spinal region, which is independent from that of related supraspinal sensorimotor structures. Moreover, a brain–spinal cord functional connectivity analysis demonstrates that the initial linear relationship between the spinal cord and sensorimotor cortex gradually fades away over the course of motor sequence learning, while the connectivity between spinal activity and cerebellum gains strength. These data suggest that the spinal cord not only constitutes an active functional component of the human motor learning network but also contributes distinctively from the brain to the learning process. The present findings open new avenues for rehabilitation of patients with spinal cord injuries, as they demonstrate that this part of the central nervous system is much more plastic than assumed before. Yet, the neurophysiological mechanisms underlying this intrinsic functional plasticity in the spinal cord warrant further investigations.

## Abstract 2

Simultaneous neuroimaging of brain and spinal cord reveals intrinsic plasticity in the spinal cord during motor sequence learning in humans, independent from that of related sensorimotor structures in the brain.

## Author Summary

When we acquire a new motor skill—for example, learning how to play a musical instrument—new synaptic connections are induced in a distributed network of brain areas. There is ample evidence from human neuroimaging studies for this high plasticity of the brain, but what about the spinal cord, the main link between the brain and the peripheral nervous system? Literature on animal models has recently hinted that spinal cord neurons can learn during various conditioning paradigms. However, human learning models by tradition assume that the spinal cord acts as a passive relay of information from the cortex to the muscles. In this study, we simultaneously acquired functional images of both the brain and the cervical spinal cord through functional magnetic resonance imaging, and we provide evidence for local spinal cord plasticity during a well-studied motor learning task in humans. We also demonstrate a dynamic change in the interaction of the brain and spinal cord regions over the course of motor learning. The present findings have important clinical implications for rehabilitation of patients with spinal cord injuries, as they demonstrate that this part of the central nervous system is much more plastic than it was assumed before.

## Introduction

Results from a plethora of studies clearly indicate that the learning of new motor skills in humans induces functional plasticity in a distributed network of brain areas [1–4]. Despite such advances in our knowledge base, the current understanding of the neural substrates mediating motor skill learning remains limited, as contribution of the spinal cord to this memory process is still unaccounted for.

Previous animal research has documented the existence of long-term plasticity in the spinal cord following basic and primitive forms of learning [5,6]. For instance, using both lesion [7] and operant conditioning [8,9] paradigms, such studies have demonstrated that motor memories can be stored within the spinal circuits after extended practice. Similarly in humans, long-term practice of skilled movements has been shown to diminish the amplitude of spinal reflexes in muscles of highly trained individuals (e.g., ballet dancers [10] and volleyball players [11]) as compared to control subjects. Yet, it is unclear whether the spinal cord manifests plastic changes during early stages of motor learning [12,13] and whether it plays an active role in the initial acquisition of new motor skills. Consequently, human motor learning models usually consider the spinal cord as a passive relay of information from the brain (controller) to the muscles (effectors), with no active learning-related role entrusted to the spinal circuitry [12]. This view mainly stems from a computational perspective of motor learning, which assumes that circuits at the spinal cord level, on which cortical plasticity is grounded and stabilized, are hardwired [6]. Contrary to this notion, new lines of evidence at the cervical [14] and lumbar [15,16] spinal levels demonstrate that spinal reflex activities can be selectively modulated by short periods of motor learning, hence suggesting that the human spinal cord may be actively involved during the learning of novel motor skills. For instance, in an innovative electrophysiological study, Meunier et al. [15] showed that homosynaptic depression in soleus muscle (i.e., a measure of local depletion in primary afferent neurotransmitters) significantly changed following adaptation to a complex pattern of changes in resistance during stationary cycling. This finding suggests that the pattern of sensory inflow plays an essential role in producing plasticity at the level of spinal cord. Despite such evidence, an important limitation of many studies using electrophysiological recordings has been that they do not allow one to distinguish plasticity in the spinal cord from functional change caused by descending cerebral inputs. Thus, whether intrinsic plasticity occurs in the human spinal cord during acquisition of new motor skills remains an open question. One way to overcome this limitation is to record both the brain and spinal cord activities simultaneously in order to assess the extent to which changes in the spinal cord activity correlate with or are statistically independent from the plastic changes that happen at the brain level during motor learning, hence allowing the identification of intrinsic changes at the spinal cord level.

**Fig 1:**
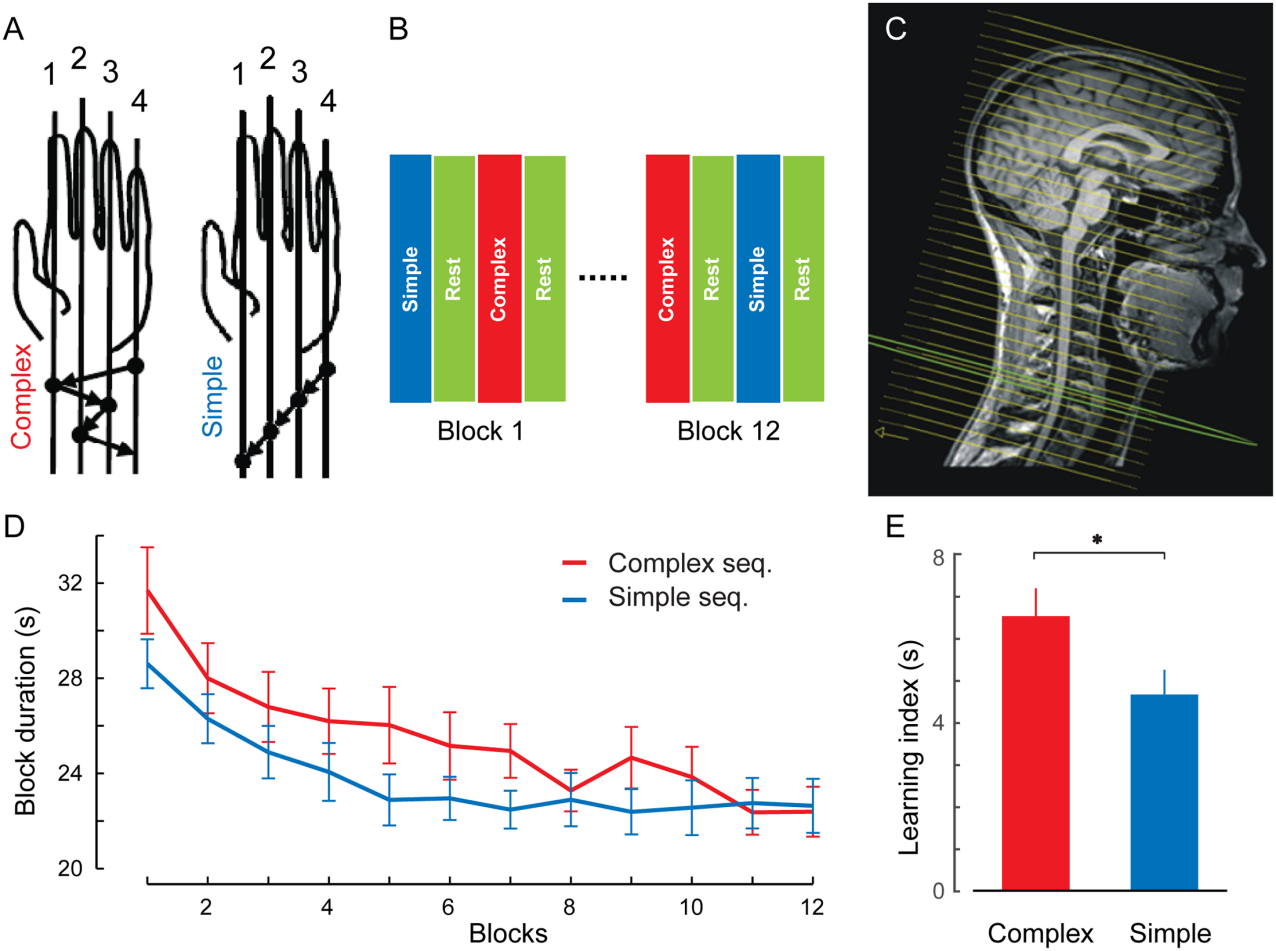
Behavioral and imaging protocols. (A) The complex (CS; 4-1-3-2-4) and simple (SS; 4-3-2-1) motor sequence learning tasks were executed with the left (nondominant) hand. Subjects were required to execute 12 CS and 12 SS blocks of practice, with 60 movements each. (B) The CS and SS conditions were split evenly across blocks and alternated in a pseudorandom fashion. A 15-s rest period preceded and followed each block. (C) Functional axial slices (displayed here over the anatomical image of a representative subject) were acquired and covered both brain and cervical spinal cord up to the first thoracic (T1) segment, and they were placed at an angle that was perpendicular to the C4 vertebral segment. (D) Performance speeds (i.e., block duration) averaged across all subjects show that the learning curves differed between the CS (red) and SS (blue) conditions. Participants reached asymptotic performance after the fourth block in the SS and after the eighth block in the CS condition. (E) Learning index (mean duration of the last two blocks subtracted from the first two blocks’ mean) revealed a significant difference in performance between the CS and SS conditions. Error bars represent standard error of the mean (SEM); * indicates *p*<0.05.

For the first time, we used functional magnetic resonance imaging (fMRI) to test the hypothesis that human spinal cord activity at the cervical level shows intrinsic learning-related changes during motor sequence learning (MSL). To do so, we acquired simultaneous images of the entire brain and cervical spinal cord during performance of a motor sequence task in healthy young subjects (Fig 1A–1C). A well-known MSL paradigm was chosen so that we could then link the novel imaging findings found here at the spinal cord level to the well-established behavioral determinants and neural correlates of MSL at the brain level (e.g., see [2,17], for reviews). This allowed us to examine the relative contribution of cortical, subcortical, and spinal regions in modulating performance during the early acquisition phase of a new sequence of movements and to investigate the functional connectivity between these structures over the course of motor learning.

## Results

### Behavioral Performance

We used a specific slice prescription approach to acquire functional scans of the whole human brain and cervical cord during performance of an MSL task (Fig 1A–1C). We trained participants (*n* = 25, median age = 24.0 y; number of males: 11) to carry out a given self-generated five-element finger sequence (Fig 1A, complex sequence [CS]) and a matched control motor sequence task (Fig 1A, simple sequence [SS]) with their nondominant left hand. They were required to perform the finger movements in the CS and SS conditions as quickly and accurately as possible and to make as few errors as possible. The CS and SS conditions were split evenly across blocks in a pseudorandom fashion and were flanked by blocks of rest periods (Fig 1B). The overall mean error rate was 3.58%, with 3.2% errors committed out of all trials during the CS and 3.95% errors during the SS condition. The Wilcoxon Signed Ranks tests indicated that there was no statistically significant difference in error rates between the two conditions (*p* = 0.56). An ANOVA for repeated measures on performance speed across all blocks in both CS and SS conditions revealed that learning curves differed across the CS and SS conditions (Fig 1D; significant condition × blocks interaction; *F*_11,264_ = 1.92, *p*<0.05). This suggests that distinct learning mechanisms were involved during the SS and CS training conditions, the former being due to a general improvement in motor performance by repeated practice and the latter resulting from sequence-related improvements in motor performance in addition to the nonspecific motor practice effect [18]. Although, subjects’ finger movements became faster by the end of training in both conditions (mean duration of the last two blocks compared to the first two blocks in the CS, *t*_24_ = 11.0, *p*<0.001, and in the SS, *t*_24_ = 7.7, *p*<0.001), there was significantly greater improvement in speed over the course of learning during the CS condition compared to the SS condition (Fig 1E, *t*_24_ = 2.11, *p*<0.05). However, the average performance speed was significantly higher in the SS condition compared to the CS condition (main effect of condition; *F*_1,24_ = 9.4, *p*<0.01).

### Neural Correlates of Motor Practice

**Fig 2:**
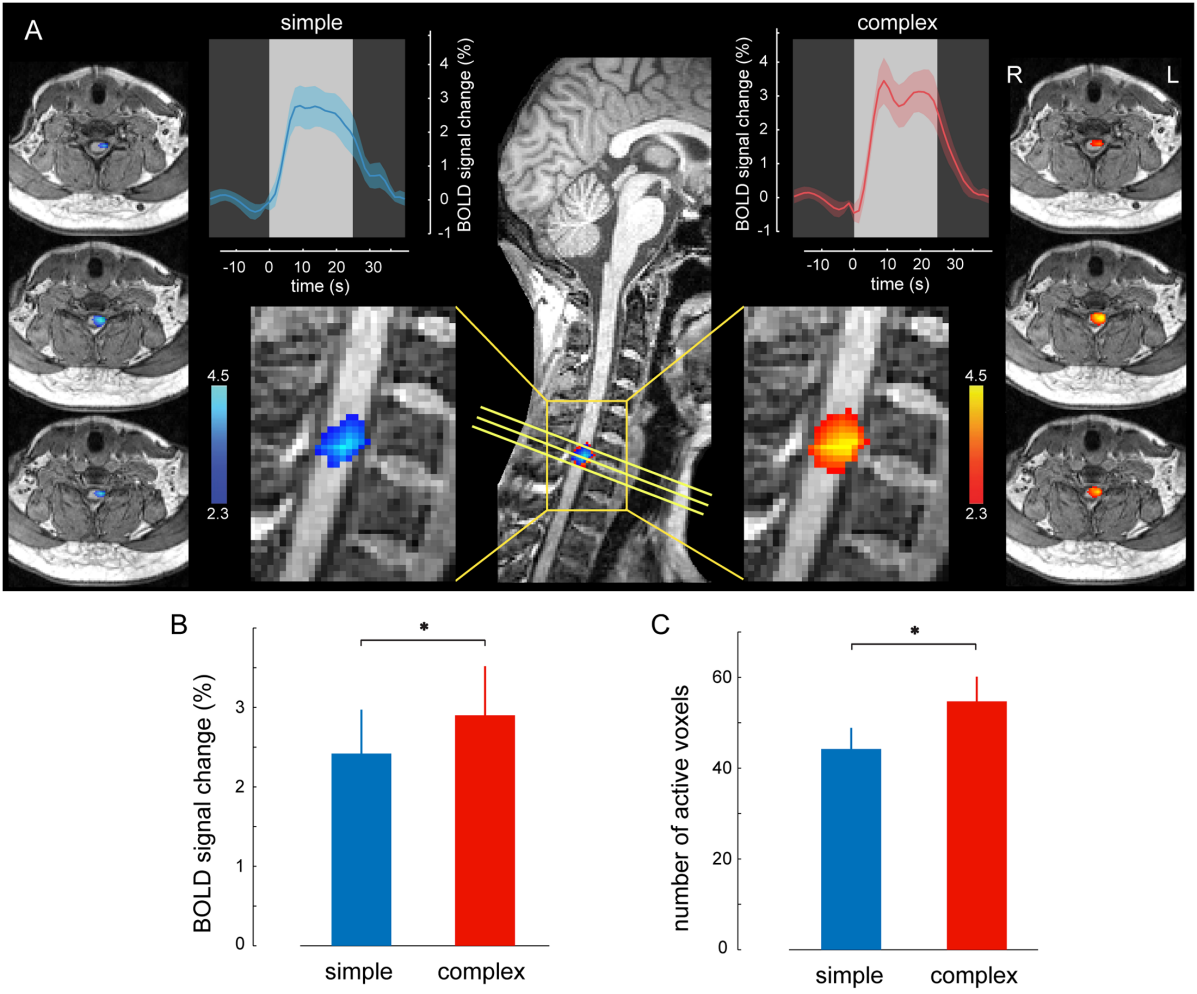
Neural correlates of motor practice in the spinal cord. (A) Activation maps representing the main effect of practice during the CS (red) and SS (blue) conditions are overlaid on the structural image of a reference subject. The yellow box indicates the sagittal section (*x* = -2.6 mm left), and the oblique yellow lines indicate the location of different transversal sections that are then displayed on the left and right sides of the figure. Note that the peaks of BOLD responses in both conditions are centered on the C7 spinal segment, mostly ipsilateral to the side of finger movements. The upper plots illustrate the percent change of the BOLD signal, averaged across blocks and subjects, during the CS (red) and the SS (blue) conditions. For averaging purposes, the BOLD signal of each block was resampled to obtain an equal number of points per block. The bright gray box represents the average duration of each block. The shaded area represents SEM; the color bars indicate *Z-*score values; all activation maps are corrected for multiple comparisons using GRF, *p* < 0.01. (B) There is a significant difference in mean amplitude of the BOLD signal change between the CS and SS conditions. (C) Similarly, the spatial extent of activation within the C6–C8 spinal segments is significantly larger in the CS as compared to the SS condition. Error bars represent SEM; * indicates *p*<0.05.

We first assessed fMRI correlates of motor practice during the SS (blue) and CS (red) conditions compared to the baseline rest periods in the brain and cervical cord separately using two repeated measures general linear models (GLM) at the group level. All the brain and spinal cord group-level activation maps were corrected for multiple comparisons using Gaussian random field (GRF) theory correction. As expected at the brain level, similar sensorimotor regions as previously reported (cerebellum, putamen, supplementary motor area, premotor and primary sensorimotor cortices) were activated during the CS and SS conditions (S1 Fig) [1,2,4]. Most importantly, however, greater blood oxygenated level–dependent (BOLD) activity was also found at the expected C6–C8 spinal levels of the cervical cord, wherein motoneurons innervating the finger muscles reside, during performance of the CS and SS conditions (Fig 2A; main effect of practice, corrected cluster-level *p*-values using GRF: *p* < 0.0001 for CS and *p* = 0.001 for SS condition). Also as expected, BOLD activity in this spinal cord region was mostly located on the side ipsilateral to the hand (left) used to perform the tasks. As shown in Fig 2A, group-level activated regions related to the main effect of practice during the CS and SS conditions were almost overlapping (i.e., peaks of activity in both conditions were located ipsilaterally at the C6–C8 spinal level). However, critically, individual subject’s activation maps related to the CS condition (S2 Fig) also revealed that 21 out of 23 subjects showed a consistent cluster of activity in that same region, hence demonstrating the robustness of this pattern of activity at the spinal cord level. Furthermore, since the activation maps were largely overlapping between the two conditions, a direct comparison between them did not result in any significant cluster at the group level using the applied family-wise corrected threshold.

### Neural Correlates of Motor Sequence Learning

To examine further the differences between the effects of CS and SS conditions (due to the acquisition of a new motor sequence and the complexity of the task itself) on BOLD changes in the spinal cord, we employed a region-of-interest (ROI)-based analysis to measure changes in the spatial extent and amplitude of the activated voxels in each participant. For each subject and condition, the ROI was selected as a set of activated voxels (Z > 2.3; *p* < 0.01 uncorrected) within the C6–C8 spinal level (see S3 Fig for the applied anatomical mask) from the individual-specific activation map of that condition. The BOLD signal amplitude changes in the CS and SS conditions were 2.9 ± 0.6% and 2.4 ± 0.5%, respectively (mean ± SEM; Fig 2B; with an average difference between the CS and SS conditions of 0.5 ± 0.18%). The average numbers of activated voxels within the C6–C8 cervical segments during the CS and SS conditions were also 55 ± 5 and 44 ± 4.5, respectively (mean ± SEM; Fig 2C). Thus, the BOLD signal amplitude and spatial extent were significantly larger in the CS condition compared to the SS condition (repeated-measure *t* test; *t*_22_ = 2.62, *p*<0.05 for amplitude and *t*_22_ = 2.57, *p*<0.05 for spatial extent). It is important to note that the latter analysis (i.e., spatial extent) was performed on preprocessed data that were not spatially smoothed in order to yield independent measures of activation amplitude and spatial extent (i.e., the larger number of activated voxels in CS was not a side effect of spread of activity into the surrounding voxels due to spatial smoothing). Furthermore, we investigated the center of gravity (mean coordinates and standard deviations) of the activated voxels in the CS and SS conditions along the different axes (*x*, *y*, and *z*) to identify the direction(s) of expansion of activity in the CS condition compared to the SS condition. Although the mean of the center of gravity across the subjects was similar in both the CS and SS conditions along all three axes (*p* > 0.35; paired sample *t* test), the spatial extent of activated voxels along the dorsoventral axis of the spinal cord was larger in the CS condition than in the SS condition (the standard deviation of the *y*-coordinate of activated voxels in each subject was on average 11% larger in the CS condition than in the SS condition; *p* = 0.055, *t* = 2.03, paired sample *t* test). Also, the standard deviation of activated voxels was, respectively, 7% and 2% larger in the rostrocaudal and mediolateral directions in the CS condition than in the SS condition, but it did not reach significance (*p* = 0.32 and *p* = 0.48, respectively).

**Fig 3:**
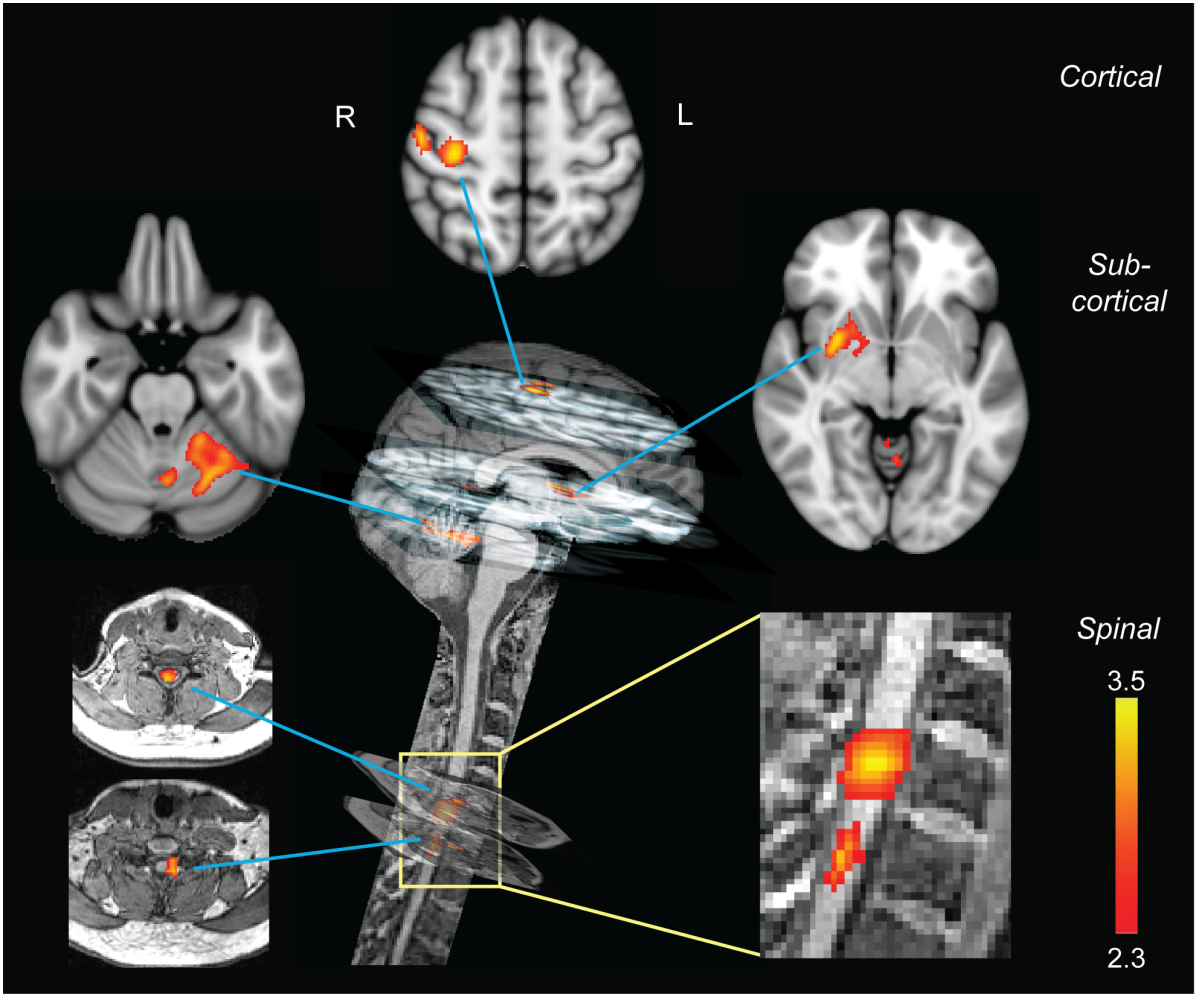
Neural correlates of motor sequence learning. Distinct cortical, subcortical, and spinal clusters showed learning-related modulation in activity only during the CS condition. All clusters of activation are positively correlated with the performance speed. At the cortical level, the activation cluster was located in the contralateral sensorimotor cortex. At the subcortical level, one cluster was found in the contralateral putamen, while the other was observed in the ipsilateral lobule V-VI of the cerebellum. In the spinal cord, activation clusters were centered on the C7–C8 spinal segments, similar to those observed in the main effect of practice. The color bars indicate *Z-*score values; all activation maps are corrected for multiple comparisons using GRF, *p* < 0.01.

To test more specifically whether the observed cervical BOLD activity was modulated by the amount of motor learning in each subject, we sought to identify the neural correlates of improvement in performance speed using a repeated-measures general linear model. The results revealed that the amount of improvement in motor performance during the CS condition, but not the SS condition, was correlated with BOLD activity changes within two spinal cord clusters located at the same spinal level as those observed in the analysis looking at the main effect of practice (Fig 3, spinal level, corrected cluster-level *p*-values using GRF: *p* < 0.01 for both clusters). Here, the activity was primarily located bilaterally in the intermediate part of C7 and in the ipsilateral C8 region. Importantly, we also investigated the difference in modulation by performance speed across conditions (speed performance by condition interaction). Similar to the results of the CS modulation alone reported above (see Fig 3), this analysis resulted again in two significant activation clusters centered at the C7 and C8 spinal levels (S4 Fig), hence suggesting that, compared to the SS condition, the CS condition generated greater modulated activity during motor learning in these particular regions of the spinal cord. Finally, it is noteworthy that the performance speed was significantly increased in both CS and SS conditions over the course of learning (p<0.001 in both condition; Fig 1D). Thus, this suggests that the lack of modulation in spinal cord activity during the SS condition could not be due to a thresholding effect. This also suggests that motor speed change alone was insufficient to account for the activation changes observed in the spinal cord over the course of learning.

Finally, we also conducted a similar analysis at the brain level using standard preprocessing and statistical methods. As expected (see [2,17]), we found clusters of activity in motor-related cortical regions including the hand-related area of the contralateral sensorimotor cortex (M1/S1 areas) and the dorsal premotor cortex, as well as two other clusters in subcortical regions including the contralateral putamen and the ipsilateral lobule V-VI of the cerebellum, all of which showed significant learning-related modulation in activity during the CS condition (Fig 3, cortical and subcortical levels, corrected for family-wise error using GRF, cluster significance threshold of *p* < 0.01; S2 Table). By contrast, and importantly, there was no brain area showing significant modulation in activity with performance speed during the SS condition.

### Distinct Spinal Cord Contribution to Motor Learning

**Fig 4:**
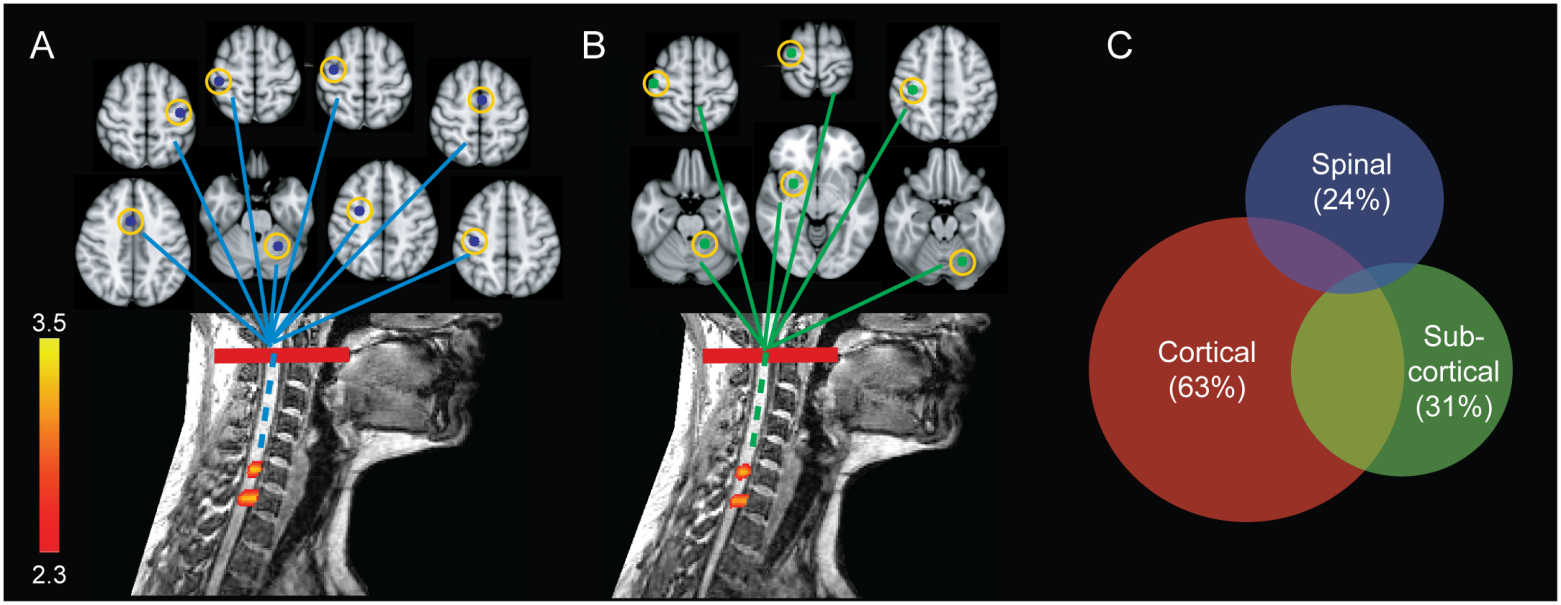
Distinct spinal cord contribution to motor sequence learning. (A and B) Two cervical clusters located at C7–C8 spinal segments showed significant changes in BOLD signal, which were modulated by performance speed. Importantly, activity in those spinal segments was independent of concomitant signals originating from both (A) brain structures that typically project to the spinal cord and (B) brain areas that show learning-related activity changes. Axial slices (colored lines) show the location of brain seed regions, highlighted by yellow circles, whose activities were regressed out in the spinal cord modulation analysis. The color bars indicate *Z-*score values; all activation maps are corrected for multiple comparisons using GRF, *p* < 0.01. (C) Activity in both the spinal cord and the brain accounted for nonoverlapping portions of behavioral variability. The Venn diagram illustrates, proportionally, the amount of performance speed variability, which is explained independently by each of the cortical, subcortical, and spinal cord ROIs, as well as their shared variance. Numbers in parentheses indicate the percentage of total variance explained by each ROI (see S3 Table).

The classical fMRI analysis employed so far showed that changes in cervical cord activity were linked to learning-related behavioral improvement, particularly in the CS condition. Yet, this approach cannot inform as to whether these changes are a simple reflection of plasticity occurring within supraspinal sensorimotor structures or whether they actually represent intrinsic local plasticity at the spinal cord level. To further explore this issue, we conducted a conditional GLM analysis based on partial correlations [19,20] to account for and partial out the possible supraspinal contribution (conditioning variables) onto the cervical cord BOLD activity (dependent variable) when modeling the learning-related improvements in behavior (independent variable). We tested two conditional models of spinal activity. In the first model, we accounted for activities of the main brain areas that are known to send direct and indirect efferent to the spinal cord, hence influencing spinal cord excitability [21–23]. These areas included the contralateral M1, dorsal and ventral premotor cortices, supplementary motor area, anterior cingulate, S1, and ipsilateral cerebellum (S1 Table). In the second model, we incorporated, as conditioning variables, all brain areas that showed learning-related changes in activity (Fig 3, cortical and subcortical levels; S2 Table). In each model, we removed the effects of activity within the conditioning brain areas on the cervical activity and investigated the remaining learning-related modulation within the spinal cord. Interestingly, the results (see Fig 4A and 4B) revealed significant changes in cervical activities located at the C7–C8 segments that were modulated in association with behavioral improvements and were independent of concomitant signals from brain structures known to project to the spinal cord (first model) or from brain areas that showed learning-related activity changes (second model), respectively. The similarity between the spinal cord activity maps estimated from these conditional models (Fig 4A and 4B) and that from the unconditional model (Fig 3, spinal level) supports the idea that the observed learning-dependent modulation in the spinal cord activity is not a mere consequence of changes in descending inflow due to cerebral plasticity, but rather is suggestive of intrinsic plastic changes that occur at the level of the spinal cord. Altogether, the results of such analyses suggest that both brain and spinal cord may assume different aspects of behavioral variability during skill acquisition.

To specifically test the latter hypothesis, we performed an analysis of variance using hierarchical regression models, which allowed us to estimate learning performance variability (dependent variable) using all possible combinations of cortical, subcortical, and spinal levels (independent variables) (see Fig 3). Hence, seven models were constructed: three based on the contribution of only one level, three using a combination of two from the three levels, and one including all three levels. The proportionate amount of performance speed variability explained by each model was assessed through an adjusted R-squared measure analysis (S3 Table). Based on the adjusted R-squared values in this hierarchical set, we then built a Venn diagram (Fig 4C) to visualize the relative influence of these different central nervous system (CNS) levels and their overlap in explaining performance speed variability over the course of learning. As shown in Fig 4C, activity in the spinal cord and the brain accounted for nonoverlapping portions of variability. Specifically, the cervical cord accounted for 24% of total explained variability, of which 81% was linearly independent from the contribution of cortical and subcortical regions in capturing performance variability during motor sequence learning (see S3 Table). This suggests that distinct neural mechanisms were responsible for the observed activity changes at the brain and spinal cord level.

### Learning-Dependent Spinal Cord–Brain Interactions

**Fig 5:**
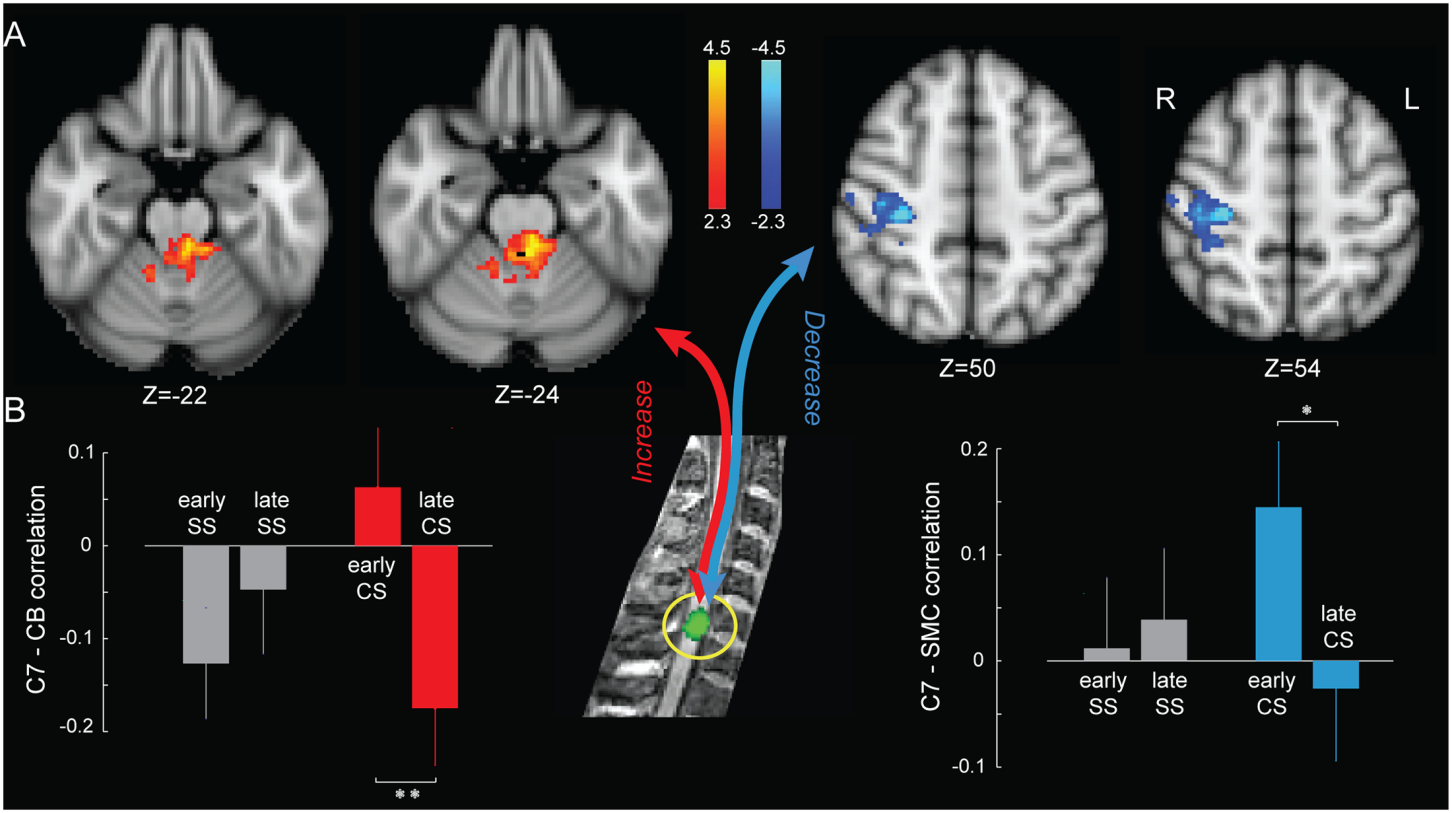
Spinal cord–brain functional interactions during motor sequence learning. (A) Activation maps show brain regions that changed their functional connectivity during the CS condition with a spinal cord ROI centered on the C7 spinal segment (yellow circle, middle of the figure). This change was proportional with subjects’ improvement in performance speed. Red and blue activation clusters indicate positive and negative relationship between functional interaction magnitude and performance speed, respectively (*p* < 0.01, corrected for multiple comparisons using GRF). (B) Bar plots show Pearson’s correlation values between the spinal cord and brain clusters’ time series in the early (the first two blocks) versus late (the last two blocks) phases of learning, averaged across subjects. Results revealed a significant increase in negative correlation with the cerebellum (CB—red bars), but a significant decrease in positive correlation with the primary sensorimotor cortex (SMC—blue bars) as learning progresses in the CS condition. There is no significant change in correlation during the SS condition (shown in gray). Error bars represent SEM; *, *p*<0.05; **, *p*<0.01, all corrected for multiple comparisons.

In order to explore whether the interaction in brain and spinal cord activity is context independent or can be altered by experience and/or learning condition, we assessed functional interaction between these structures over the course of learning using psychophysiological interaction (PPI) analyses (Eq 1) [24]. An individual-specific spinal cord ROI, centered on the C7 cervical level (see S3 Fig for the applied ROI’s mask), was selected as a seed region based on the main effect of practice during both CS and SS conditions (Fig 2A). We evaluated the changes in functional connectivity between the spinal ROI and all the brain voxels, in proportion to the subjects’ improvement in performance speed during both CS and SS conditions. It is important to note that such analysis did not reveal any significant change in brain/spinal cord connectivity during the SS condition. By contrast, a cluster in the right primary sensorimotor cortices showed a significant decrease in positive correlation with the cervical cord in proportion to the amount of learning during the CS condition (Fig 5A, right panel, contralateral M1 and S1 hand area, *p* < 0.01, corrected for multiple comparisons using GRF). Furthermore, we observed an increase in negative correlation between the spinal cord ROI and a cluster mainly located in the left anterior cerebellum in proportion to the amount of learning during the CS (Fig 5A, left panel, lobule IV and superior cerebellar peduncle, *p* < 0.01, corrected).

Finally, to investigate the amplitude and direction of functional connectivity at different stages of learning compared to baseline, we evaluated the linear correlation between BOLD signals of the reported brain areas and the spinal cord ROI in both early (the first two blocks) and late (the last two blocks) training periods during SS and CS conditions (Fig 5B). This analysis confirmed the presence of training-induced changes in BOLD signal synchronization between the spinal cord and brain, which depended upon the acquisition of a new motor sequence (repeated measures ANOVA, significant interaction between learning condition (CS versus SS) and time (early versus late), *p* < 0.05 for M1/S1, and *p* < 0.01 for cerebellum). Also, it shows that, as learning proceeds throughout CS condition, the activity within the cervical ROI becomes less correlated with that of primary sensorimotor cortex (*t*_22_ = 2.8, *p* < 0.05, corrected) but becomes more negatively synchronized with that of the anterior cerebellum (*t*_22_ = 3.3, *p* < 0.01, corrected).

### Sequence-Specific versus Nonspecific Changes in Motor Output

In order to examine the possibility of nonspecific changes (unrelated to the MSL process) in motor output during the course of learning, we measured the power of electromyography (EMG) signals during training in both CS and SS conditions. A separate control group of subjects (*n* = 10, median age = 23 y; number of males = 5) was recruited and underwent the same motor learning procedure as the experimental group described above, except that testing was carried out in a magnetic resonance scanner simulator while recording EMG signals from several related extrinsic (flexor digitorum superficialis and extensor digitorum) and intrinsic (first dorsal interosseous and flexor digiti minimi) hand muscles. This allowed us to test for any motor execution-related differences at the periphery, which might be causing the pattern of spinal cord activity differences observed across conditions. The motor learning curves, as measured by speed performance in the control group, were very similar to that of the experimental group (S5 Fig; mean duration of the first two blocks in the control group compared to the experimental group during the CS: *p* = 0.77 [two-sample *t*-statistics, *df* = 33], and during SS: *p* = 0.57; and mean duration of the last two blocks during CS: *p* = 0.75, and during SS: *p* = 0.89). EMG analysis during the CS and SS conditions did not reveal any significant effect of condition on the normalized EMG power or root-mean-square (RMS) in any of the tested muscles (main effect of learning condition: *p* > 0.14 in all comparisons; S6 Fig). Also, the normalized EMG power (and RMS) was not statistically different over the course of training between CS and SS conditions (condition × block interaction: *p* > 0.2, *df* = 11). These results suggest that the observed plastic changes at the spinal cord level were not a by-product of variations in the applied muscle force (as estimated by EMG power) during motor learning.

## Discussion

For the first time, to our knowledge, we simultaneously acquired functional scans of the human brain and cervical cord during performance of a motor sequence learning task. Using this procedure, we show that learning a complex motor sequence, as compared to a simple one, produces greater BOLD activity (both in amplitude and spatial extent) within the C6–C8 levels of the ipsilateral spinal cord. Furthermore, the activity within this cervical cluster is modulated in association with behavioral improvements in performance, and significantly more so during practice of a complex sequence compared to that of a simple one. Importantly, we also provide strong evidence for local spinal plasticity over the course of human motor learning through a combination of partial correlation and hierarchical regression analyses. Finally, we demonstrate that the spinal cord becomes less synchronized with cortical sensorimotor areas and more negatively correlated with the anterior cerebellar cortex as learning progresses. The latter findings suggest that, over the course of motor learning, there is a decrease in the linear relationship between the cervical cord and primary sensorimotor cortex, while the inhibitory connection between the spinal cord and cerebellum gains prominence.

### Challenges in Spinal Cord Imaging and Analyses

Compared to brain functional neuroimaging studies, there is a scarcity of experiments that aimed to scan the activity of the spinal cord. This can be explained by the fact that imaging the spinal cord raises several challenges. These stem primarily from the small size of this structure, the magnetic field inhomogeneities caused by the many different tissue types surrounding it, and the movements induced by respiration [25,26]. Accordingly, in the present study, we undertook a series of steps, both during data acquisition as well as analyses, in order to overcome these challenges and to ensure that activations detected in the spinal cord were genuine and not the results of various artefacts. During data acquisition, we first used a slice prescription pulse sequence that yielded at least one good-quality slice per cervical level and minimized the inhomogeneity due to the surrounding tissue. Second, we acquired gradient-echo field map images to estimate field inhomogeneities. Third, we used an in-plane spatial resolution of 2.5 mm^2^, hence allowing there to be at least 12 voxels covering the spinal cord while maintaining sufficient signal-to-noise ratio (SNR). Furthermore, during data preprocessing, we first used the acquired field map images to correct for field distortions and carried out an independent component analysis to extract and remove the cardiac and respiratory-related physiological noise components from data based on their spatiotemporal characteristics. Second, we then estimated the spinal motion parameters (three rotations and three translations) and excluded from analysis the volumes in which the spinal cord displacement was higher than 1 mm. Accordingly, the number of deleted volumes ranged from 0 to 14 with a median of 2 per subject out of the total number of acquired volumes, which varied from 283 to 453 depending on participant’s execution speed (mean of 384.2 and median of 386 volumes). Third, we used the motion parameters as confounds in the GLM analysis to account for motion artefacts.

Other important arguments against the possibility that our results reflect motion artifacts are (a) the fact that block-related averages of the spinal BOLD signal (Fig 2A, inlets), which are model free and not convolved by the hemodynamic response function (HRF), showed the typical 8-s delay from the block onset until they reach the plateau and (b) that we also defined subject-specific functional masks that included two additional voxels outside the spinal cord in each direction, both along the anteroposterior and mediolateral axes (S7 Fig), hence allowing the detection of spurious activity outside the spinal cord confines, if any. At the end, our results were robust, as all detected activity peaks were focal, within the spinal cord boundaries, and were located at the expected site of the spinal cord (mostly ipsilateral and centered on C7–C8), in line with the location of motoneurons innervating the finger muscles involved in the motor task. However, because of the large magnetic inhomogeneity produced by the intervertebral disks (Fig 1C), our analysis is unable to determine whether the two activated clusters in the rostrocaudal plane at the C7 and C8 cervical levels (Fig 3) are part of the same functional unit (seemingly split because of the presence of a low SNR slice at the disk level) or whether they actually represent two functionally distinct clusters. Yet, when lowering the statistical threshold to *Z* = 2, we observed that the two clusters joined together, hence possibly supporting the former case. Despite this uncertainty, however, our results strongly suggest that spinal activity was not a mere reflection of the speed with which subjects executed the finger movements; even though movements in the SS condition were faster, the spatial extent and amplitude of the spinal activation were greater during the CS condition. Importantly, in counting the number of activated voxels, we did not perform any spatial smoothing in order to prevent the spread of activity from surrounding voxels, hence yielding independent measures of activation amplitude and spatial extent (although the partial volume effects in BOLD fMRI acquisition can cause some potential confounds in this analysis). Lastly, spinal cord activity was modulated in association with behavioral measures of performance speed in the CS condition only (and not the SS condition), hence supporting a process that is associated with both the complexity of the motor sequence and improvements in performance during learning.

### Methodological Considerations and Contributions

Because we were interested in acquiring data covering both the brain and spinal cord, the in-plane spatial resolution afforded in our present fMRI study was somewhat limited (2.5 x 2.5 mm^2^ in-plane). Considering the small size of the spinal cord, this makes it difficult to draw a clear-cut conclusion on the precise location of focal task-related activity within the cervical cord. Nevertheless, because of the larger size of the cervical cord along the mediolateral versus anteroposterior axis, we believe that the resolution used here allowed us to be relatively confident about the ipsilaterality of the focal activation, and less so about the dorsal/ventral localization of the activity. Accordingly, we would argue that the peaks of activity corresponding to the main effect of practice during both CS and SS conditions (Fig 2A), as well as to the learning-related activity in the CS condition, were mostly located on the ipsilateral side of the cervical cord in line with the hand used during the motor task and the known spinal cord anatomy. Our study brings an important methodological contribution to the field of motor learning through the fact that the present pattern of functional motor learning-related cervical plasticity was observed for the first time using simultaneous brain/spinal cord fMRI in a large group of subjects. The innovative way of making use of the extensive field of view of the Siemens TIM Trio MR system (50 cm in the rostrocaudal plane) in the current study allowed us to investigate quantitatively functional interactions between those structures and get insights into the neural substrates mediating motor sequence learning at all levels of the central nervous system. To acquire such data through imaging of both brain and spinal cord, some researchers have previously used custom-made coils [27] or dedicated imaging pulse sequences [28]. Opening the field for future studies, the present study employed regular equipment and sequences provided by the manufacturer, hence facilitating further the generalization of the imaging methods used here. Finally, contrary to most of the previous spinal imaging work that has reported activation maps at the subject level only, with the exception of a few reports on spinal cord modulation of pain [29,30], the present study reports group data, because we developed an analysis procedure that allowed us to overcome several challenges, including those related to image normalization and alignment, a low SNR that limits the power of statistical analyses, and the physiological noise inherent to functional imaging of the spinal cord.

### Learning versus Execution

A limitation of the current study is the extent to which motor learning can be decoupled from performance speed changes, given that the performance speed measure itself is an indicator of both learning and better motor execution. To address this issue, we used a control task (SS), in which subjects knew the sequence very well, but performance speed was significantly increased over the course of training. Importantly, no modulation in the spinal cord activity was identified with respect to performance speed during this control task. Furthermore, the present pattern of results does not appear to be related to a possible confounding factor like differences in force used to perform the two tasks, as an independent study investigating EMG activity associated with learning did not reveal any significant disparities in EMG power (measured by root-mean-squared values), which is a good estimate of applied muscle force during voluntary contractions.

Finally, another means previously used to investigate the effects of learning, nonconfounded by unspecific changes due to task execution, has been to conduct functional connectivity analyses of resting state data [31]. Interestingly, this approach has recently been performed successfully using BOLD fMRI in the spinal cord [32,33]. However, the experimental design employed in the present study did not allow us to carry out such analyses because of the limited number of acquired volumes during rest periods between blocks of task-related activity. Thus, our results do not permit us to determine whether the task-based functional changes observed at the cervical level persisted after practicing the learning task, a finding that awaits further investigation.

### Spinal Cord Imaging of Motor Behavior

In line with early seminal work by Yoshizawa and colleagues [34], there has been increased interest in recent years in investigating spinal cord BOLD activity during the execution of simple motor tasks [35–38]. While these studies have demonstrated the feasibility of BOLD spinal cord imaging during simple motor performance, none have examined the neural correlates of motor learning in the spinal cord or acquired simultaneous functional images of the brain and spine during performance of a motor learning task. Using another contrast mechanism based on proton density, called signal enhancement by extravascular water protons (SEEP) [39], other researchers have also found strong motor- and sensory-related activity within the spinal cord. Recent studies comparing the BOLD and SEEP mechanisms have also consistently shown comparable results using either of these contrasts [37]. Yet, the present results expand our understanding of motor skill acquisition by showing that the spinal cord is an integral part of the neural network involved in this process. Until now, models of motor sequence learning in humans have elegantly described the experience-dependent plasticity that occurs in brain regions at different phases of the acquisition process but have not assigned any functional plasticity at the spinal cord level. At the cerebral level, the early stage of learning is supported by widespread activations predominantly in both the corticostriatal and corticocerebellar networks [1,2]. Our findings, however, show that in addition to these two systems, learning a new sequence of movements also includes plasticity within the cervical spinal cord. Furthermore, our results reveal the presence of intrinsic functional plasticity in the spinal cord that is associated with learning-related changes in motor performance and that is linearly independent from that of supraspinal structures. Although this does not exclude the possibility of a nonlinear relationship between activities in cervical and sensorimotor brain regions, it does eliminate the simplest possible alternative interpretation, which is that learning-related changes in cervical activity are merely mirroring the ongoing higher-level cerebral plasticity. Altogether, our results thus lend strong support to the idea that intrinsic plasticity can be induced at the spinal cord level in the early stages of motor learning.

### Spinal Cord Contributions to Motor Learning

Although still conjectural, two possible reasons may explain the spinal cord’s active role during motor skill acquisition. First, together with learning-induced cortical and subcortical plasticity, the spinal cord could be another site where motor memories are stored. Previous studies have found that different types of motor memories can be encoded at the spinal level. For instance, animal research has shown that central pattern generators can be recovered in partially or totally transected cord following locomotor training [7]. In human studies with intact spinal cord, long-term changes in Hoffman reflex (H-reflex) in highly skilled individuals as compared to nonskilled individuals have also been reported, hence suggesting that the spinal cord is capable of encoding local motor memories [10,11]. A second reason may involve the stabilization and facilitation of movement execution through modification of muscle and joint stiffness as the motor skill is being acquired during practice. One possible way that such a process is achieved might be through elevated cocontraction of antagonistic muscles, which is known to occur during early stages of motor learning [40]. Indeed, muscle cocontraction levels can be directly adjusted via inhibitory mechanisms (e.g., presynaptic or disynaptic inhibition) at the spinal cord level [10]. In line with this view, we have previously demonstrated that the H-reflex, a local measure of excitability of sensorimotor pathway within the spinal cord, was systematically diminished over the course of motor sequence learning, in which the reduction in reflex amplitude was greater compared to a control simple sequence or random movements [14]. It has been suggested that this persistent decrease in the H-reflex amplitude could be the result of increased presynaptic inhibition of the Ia afferent transmission to motoneurons [8,10]. Thus, the increased local cervical activity over the course of learning observed in the current study might be related to such synaptic mechanisms responsible for the reduction of the H-reflex at the spinal cord level. While our current findings cannot distinguish between these two functions of the spinal cord in motor learning, they certainly give support to the idea that spinal cord plays an active, rather than passive, role in this process. Furthermore, the scanning paradigm used in the present study offers a necessary tool for future investigations that specifically aim to parse out the roles of the brain and the spinal cord in various stages of motor learning.

As verified by our EMG analysis, the motor task in the current study required both activations of extrinsic and intrinsic hand muscles, which are influenced by both indirect and direct corticomotoneuronal connections [41,42]. It is thus conceivable that motor learning could also be associated with functional changes in spinal circuitry involving both the interneurons as well as motoneurons, as illustrated by the fact that cervical activations detected in our study were not exclusively confined to the ventral horn of the spinal cord.

### Possible Mechanisms Underlying Changes in Brain/Spinal Cord Interaction

Interestingly, connectivity analyses revealed that activity within the spinal cord was functionally more synchronized with the cerebellum, but less synchronous with primary sensorimotor cortical areas as learning progressed. Several possible mechanisms might explain the observed pattern of connectivity between the brain and spinal cord. First, the decreased interaction between the spinal cord and sensorimotor cortical areas might reflect the reduced one-to-one control of individual muscles by sensorimotor cortex late in the learning, through processes such as chunking [43] or the recruitment of muscle synergies [44]. In the case of muscle synergies, for example, various models have posited the existence of a module relying on spinal interneurons that generates a specific pattern of muscle activation [44]. Thus, as learning of sequential movements progresses, it is conceivable that the development of new muscle synergies leads to a greater local functional integration within the spinal cord and to a reduction in the one-to-one linear relationship with sensorimotor areas, hence resulting in a reduced linear functional connectivity between the spinal cord and sensorimotor cortex later in the acquisition process. A second possible mechanism could be that there was a shift in attentional focus as learning progressed [45], which could then explain the functional decoupling between the sensorimotor cortex and spinal cord. In fact, the latter hypothesis is supported by the reported alteration of corticospinal excitability induced by attentional modulation during the performance of a motor task [46]. Finally, another probable mechanism could rely on corticospinal inhibitory processes. Although there are no direct long-range inhibitory corticospinal projections, the learning-related decrease in functional connectivity between these two structures might be the result of an increase in the weight of supraspinal projections onto the inhibitory spinal interneurons, which then project to ventral horn motoneurons [47]. Regardless of the mechanism of action, which cannot be directly tested with fMRI, the results of our functional connectivity analysis reveal that, at the end of learning, there is no significant correlation between cortical and spinal cord activities, indicating that the two structures become functionally desynchronized as learning progresses.

By contrast, the increase in negative synchronization between activity in spinal cord and cerebellum could be more likely related to inhibitory mechanisms. Indeed, several electrophysiological studies have reported that cervical cord activity is inhibited when stimulating anterior medial neurons of the cerebellar cortex [48,49]. As the cerebellum is particularly involved in the coordination of rhythmic and oscillatory movements [50], such increased interaction (in the form of an inhibitory effect) between the cerebellum and cervical cord over the course of motor sequence learning might reflect the underlying neural mechanisms responsible for the temporal control of rhythmic finger movements [51]. On the other hand, another possibility that might explain these results is that the cerebellum is implicated in controlling muscle and joint stiffness by adopting optimal strategies [52], which in turn can be achieved through various spinal inhibitory mechanisms, as explained above.

### Conclusion

In sum, we believe that the simultaneous imaging and analysis of motor functions of the brain and spinal cord in this study can benefit the neuroscientific community, which has so far been divided in its investigation of the neural substrates mediating neuroplasticity in only one of these two structures. The present findings also have important clinical implications for the rehabilitation of patients with spinal cord injuries, as they demonstrate that this part of the central nervous system is much more plastic than it was assumed before. Altogether, the present findings support the view that the cervical spinal cord plays a critical role in our ability to acquire new motor skills, although the neurophysiological mechanisms underlying such neural plasticity await further investigations.

## Materials and Methods

### Participants

Twenty-five healthy young adults (14 females, 11 males; median age: 24 y) participated in the present neuroimaging study. A separate group of healthy young adults (*n* = 10, 5 females; median age: 23 y) participated in the EMG recording/motor learning experiment. All participants were right-handed, as determined by the Edinburgh Handedness Inventory. The study was approved by the joint research ethics committee of the Regroupement Neuroimagerie Québec at the Centre de recherche, Institut universitaire de gériatrie de Montréal, which follows the policies of the Canadian Tri-Council Research Ethics Policy Statement and the principles expressed in the Declaration of Helsinki. All participants gave their informed written consent.

### Finger Motor Sequence Learning Task

Subjects were tested using a version of the MSL task [53], in which they were required to perform self-generated finger movements with their nondominant (left) hand as quickly and with as few errors as possible. Prior to the experiment, subjects practiced the sequential movements briefly (up to three correct consecutive repetitions per sequence) via an fMRI compatible button box. During the experiment, however, subjects laid supine in the scanner and executed the task following written instructions, which appeared on a screen visible via a mirror attached to the head coil. During “Rest,” subjects had to rest with their eyes open for as long as the instruction appeared on the screen. When the instruction “Sequence” appeared, participants had to execute repeatedly a five-element motor sequence (4-1-3-2-4; complex sequence condition [CS]; where digits 1 to 4 correspond to the digits of the left hand from 1 [the index finger] to 4 [the pinkie]). Finally, when the word “Control” appeared on the screen, participants were required to execute a simple, four-element sequence repeatedly (4-3-2-1; simple sequence condition [SS]). In total, subjects were administered 24 blocks of 60 movements each (corresponding to 12 or 15 repetitions of the complex and simple sequence per block, respectively), separated by rest periods lasting 15 s each. The two experimental conditions were split evenly across blocks (i.e., 12 blocks each) and alternated in a pseudorandom fashion across blocks (Fig 1B), with no more than two consecutive blocks in the same experimental condition. The total task duration varied between 11 min and 48 s to 18 min and 53 s, with a median of 16 min and 6 s, depending on participant’s speed of movement execution. Reaction time (time elapsed between two consecutive key presses), block duration (time to accomplish each block), and errors (number of incorrect key presses in each block) were recorded.

### Imaging Parameters

Images were collected using a 3T whole-body Siemens TIM TRIO scanner with simultaneous detection via the four-channel neck, 12-channel head, and 24-channel spine coils. A structural volume was acquired in the sagittal plane using a magnetization-prepared rapid gradient echo (MPRAGE) sequence (TR = 2,300 ms; TE = 3.31 ms; FoV = 320 × 320 mm; matrix size = 256 × 256; 160 slices, slice thickness = 1.3 mm, in-plane resolution = 1.25 × 1.25 mm). For functional acquisitions, an echo-planar imaging (EPI) gradient echo sequence was used with the following parameters: TR = 2,500 ms; TE = 30 ms; FA = 90°; FoV = 160 × 160 mm; matrix size = 64 × 64; slice thickness = 4 mm; in-plane resolution = 2.5 × 2.5 mm, parallel imaging with an accelerated factor of 2 and GRAPPA reconstruction. In total, 35–37 transversal slices per volume were acquired, as described in a previous study [54]. Coverage of the cervical cord of each subject was achieved by recording functional data from the 15 axial slices spanning the C1 to C7 cervical vertebrae (corresponding to the C1 to C8 cervical spinal segments). Slices were spaced from 80% to 120% of the slice thickness in order to cover both the brain and the cervical spinal cord up to the first thoracic (T1) segment, and they were placed at an angle that was perpendicular to the C4 vertebral segment in order to get the best in-plane coverage of the spinal cord (Fig 1C). Slices were centered alternately at the midvertebral body level and at intervertebral disks [54]. The variation in the number of slices across subjects was due to the intersubject differences in gross spinal and brain anatomy. This particular slice prescription ensured that, despite individual anatomical variations, each cervical segment was covered using an axial slice passing through its center, hence making possible the precise coregistration of the functional data across participants. The number of acquired functional volumes was variable, depending on the participant’s speed during the task. Finally, dual echo field map images (TE1 = 4.92 ms, TE2 = 7.38 ms) were acquired to correct for the susceptibility-induced geometrical distortions in EPI data.

### Brain Data Analyses

Image processing was carried out using the FSL software package [55] and in-house programs developed in MATLAB. First, each functional volume for each subject was split into the brain and the cervical cord in the inferosuperior direction. The segmented brain functional images were first processed using regular preprocessing steps including motion correction, high-pass temporal filtering (σ = 100 s), non-brain-tissue removal using the Brain Extraction Tool (part of FSL), spatial smoothing (6 mm Gaussian kernel), and registration to the Montreal Neurological Institute (MNI) standard space. For each subject, changes in brain regional responses were estimated using a model including responses to the task practice conditions (CS and SS) and their linear modulations by performance speed (negatively correlated with block duration). These regressors consisted of boxcars convolved with a double-gamma HRF. Six rotation and translation motion parameters were also included in the model as confounds. Modulation by performance speed identified regions where response amplitude changed as motor behavior became faster across blocks of practice. The subject-level regression coefficients and their covariance maps were then input to a group-level analysis, which used a mixed-effects general linear model (Z > 2.3, corrected family-wise error using Gaussian random field theory, cluster significance threshold of *p* < 0.01).

### Spinal Cord Data Analyses

The preprocessing pipeline for analyzing the spinal cord functional image comprised the following: (a) creating a mask of the cervical cord by visual inspection—the mask included at least two additional voxels outside the spinal cord on the left, right, dorsal, and ventral sides (S7 Fig); (b) correcting for motion; (c) removing volumes with an absolute motion value > 1 mm from the target image; (d) utilizing GRE field map unwarping to correct for geometrical distortion (S8 Fig); and (e) applying high-pass temporal filtering (cutoff period = 100 s). Two subjects were excluded from further analysis because of excessive movement during fMRI scanning. We generated two datasets with different spatial smoothing parameters (0 and 6 mm Gaussian kernel), which were used in two separate general linear models analyses in order to estimate task-based activity at the group level (see below). Preprocessed data with no smoothing were used in the PPI analysis to calculate the BOLD signal average within the ROI, as explained below. Furthermore, smoothing was performed inside a mask within the cervical cord to ensure there was no infiltration of the surrounding structures’ signals into the cervical BOLD signal.

Manual registration was performed in order to align EPI cervical slices of every subject to the EPI cervical image of one of the subjects, which was selected as the template. To do so, for each subject the center of cervical cord in each slice was manually marked and then shifted in the lateral and anteroposterior axes to match that of the template (S8 Fig). Because of the subject-specific prescription of axial slices and slice gap-size adjustments, the cervical EPI slices were already aligned along the rostrocaudal axis across subjects. As physiological noise has a major impact in the detection of spinal cord BOLD signal [26], we then used independent component analysis to identify and account for noise components at the cervical cord level [56]. Thirty components were extracted for each subject, which accounted for about 95% of the BOLD signal variability. The components that met the following criteria were considered as noise: (i) the component’s time series had more power at high frequencies (more than 50% of power was associated with frequencies larger than 0.08 Hz [31]; task frequency was around 0.02 Hz) and (ii) the component’s spatial map showed more activated voxels outside the spinal cord (more than 50% of significantly activated voxels [Z > 2.3] were outside the spinal cord mask). Overall, 4 to 12 components met both criteria in every subject, and their time series were thus included as confound in the subject-level general linear model analysis.

For each subject, the preprocessed cervical BOLD responses were estimated using a model that included the task practice conditions (CS and SS) and their linear modulations by performance speed (normalized to Z-scores with a standard deviation of one and a mean of zero), as well as nuisance regressors comprising the six rotation and translation motion parameters, the time series of noise components extracted from the independent component analysis, and the average of white matter and CSF signals extracted from the brain as described previously [31]. By normalizing the performance speed regressors to Z-scores, their GLM coefficients in each condition became independent of the absolute differences in motor speed across conditions; they rather reflected the relationship between normalized performance variability within each condition and BOLD signal change in the spinal cord. The subject-level regression coefficients and their covariance maps were then input into a group-level analysis, which used a mixed-effects general linear model [55]. The corresponding *Z*-statistics maps for the contrasts of interest were generated and corrected for multiple comparisons using Gaussian random field theory (minimum Z > 2.3; cluster significance threshold, *p* < 0.01, corrected). Furthermore, in order to compare the spatial extent of cervical activation across conditions, we identified all of the activated voxels (Z > 2.3; *p* < 0.01 uncorrected) within the expected spinal levels (C6–C8; segments wherein motoneurons innervating the finger muscles reside) based on the subject-level activation maps in each condition. In this analysis, we used the preprocessed data that were not spatially smoothed (smoothing kernel of zero) to ensure that the number of activated voxels count was not confounded by the spread of activity to the neighboring voxels caused by spatial smoothing. We then calculated the percent BOLD signal change averaged over voxels that were activated in both CS and SS conditions (conjunction map). In order to compare the amplitude of cervical activation across conditions, we evaluated the percent signal change in the conjunction map in each block of practice for each subject.

### Conditional Spinal Cord-Brain Activity Analysis

A simple technique based on the notion of partial correlation [19,20] was used to account for the possible supraspinal contribution to the spinal BOLD response when modeling the learning-related effects. For each subject, the cervical BOLD responses were again estimated using a model that included the task practice conditions (CS and SS) and their linear modulations by performance speed (learning-related regressors), but this time we included the time series of *n* conditioning brain areas *x*_*1*_, *x*_*2*,_…, *x*_*n*_ as covariates. The design matrix also included the time series of nuisance signals described earlier. The learning-related regressors were orthogonalized with respect to all confound variables; this is mathematically equivalent to subtracting and removing mutual dependencies of the conditioning brain areas from the spinal cord activity when estimating the effects of learning [20]. Two conditional models were tested. In the first model, we incorporated, as confound, all the main brain areas that are known to send efferent information and thus that can influence spinal cord excitability [21–23] (i.e., contralateral M1, dorsal and ventral premotor cortices, supplementary motor area, anterior cingulate, S1, ipsilateral M1 and cerebellum). For each region, the peak of activity from the main effect of practice during both CS and SS conditions was extracted as seed voxel (S1 Table). In the second model, we incorporated, as confound, all brain areas that showed sequence learning-dependent modulation. The seed voxels for this model were extracted from the peaks of activity using performance speed as a variable of interest (i.e., parametric modulator), when modeling brain BOLD response during CS condition (including M1, dorsal premotor, S1, putamen, and lobule V–VI of the cerebellum; Fig 3 top panels; S1 Table). For each subject and seed voxel, we calculated the average of the BOLD signal in a standard spherical mask (radius = 6 mm) around the seed.

### Hierarchical Regression Analysis

In order to quantify the relative contribution of the spinal cord and the brain to behavioral improvements during motor sequence learning, we measured the explained variance in hierarchical general linear models. Each regression model estimated behavioral improvements (as measured by performance speed convolved with HRF) using a design matrix that included the time series of different brain and spinal cord areas. To obtain these time series, we selected all activation clusters, either in the brain or the spinal cord, which were modulated by the performance speed (Fig 3; S2 Table), including the sensorimotor cortex (cortical level), putamen and cerebellum (subcortical level), and C7 cervical segment (spinal level). We then used principal components analysis in combination with an adaptive pruning algorithm [57] to estimate the number of non-noise components in each cluster of interest. On average, nine components per cluster were extracted for each subject. We then constructed seven hierarchical regression models using different combinations of cortical, subcortical, and spinal levels (only cortical, only subcortical, only spinal, cortical + subcortical, cortical + spinal, subcortical + spinal, and cortical + subcortical + spinal). For each model, we calculated the adjusted R-squared value (adjusting for the number of predictors in each model), which indicates the proportionate amount of variation in the performance speed explained by the time series of components from different levels (S3 Table).

### PPI Analysis

PPI analyses [24] were performed to test the functional connectivity of the brain with a reference spinal cord ROI, in proportion to performance speed changes during practice. New generalized linear models were constructed at the individual level, using several regressors to model brain activity at each voxel over time (***y(t)***):

$$y=\left[\beta_{1}\ldots \;\beta_{7}\right]\;{\left[x_{1}\ldots x_{7}\right]}^{T}+\left[\beta_{8}\ldots \;\beta_{15}\right]\;{\left[c_{1}\ldots c_{8}\right]}^{T}+\varepsilon$$ (1)

Four regressors represented the main effect of practice in each condition (***x***_***1***_ and ***x***_***2***_) and their modulation by performance speed (***x***_***3***_ and ***x***_***4***_). The fifth regressor was the mean activity in the spinal cord ROI (***x***_***5***_; physiological regressor, see below). The last two regressors of interest represented the interactions between each of the psychological (modulation by speed performance during CS and SS) and the physiological regressors (i.e., ***x***_***6***_ = ***x***_***3***_. ***x***_***5***_ and ***x***_***7***_ = ***x***_***4***_. ***x***_***5***_). The psychological regressors were convolved with HRF. The design matrix also included movement parameters (***c***_***1***_–***c***_***6***_), as well as average CSF (***c***_***7***_) and white matter signals (***c***_***8***_) as confound. The reference spinal cord ROI was selected based on both anatomical (expected cervical level) and functional (task-related) constraints in the functional space of each subject, in order to attain high sensitivity by selecting individual-specific spinal cord activation maps. For each subject, all the activated voxels in the conjunction maps between CS and SS main effect of practice (Z > 2.3; p < 0.01 uncorrected) within the C6–C8 spinal levels (see S3 Fig) were selected as ROI. A significant PPI indicated a change in the strength of the functional connectivity between any reported brain area and the spinal cord ROI, which was related to performance speed changes during practice. To examine the direction of functional connectivity changes over the course of learning, we then evaluated the correlation between the BOLD signals of the reported brain areas and the spinal cord region of interest early (the first two blocks) and late (the last two blocks) during training in each of the SS and CS conditions.

### EMG Recordings

In a control experiment, ten young healthy adults were recruited and underwent the same motor learning protocol as the main experimental group. No brain imaging was performed, but in order to mimic the experimental environment and positioning of the subjects as much as possible, the experiment was done in a mock scanner at the Functional Neuroimaging Unit, Montreal. Subjects laid supine in the mock scanner and executed the task following written instructions, which appeared on a screen visible via a mirror. Similar to the experimental group, subjects performed 24 blocks of 60 movements each (12 in CS, 12 in SS, pseudorandom alternation), separated by 15-s rest period blocks. Additionally, EMG signals were recorded from two extrinsic (flexor digitorum superficialis [FDS], and extensor digitorum [ED]) and two intrinsic (first dorsal interosseous [FDI], and flexor digiti minimi [FDM]) finger muscles during the experiment. FDS and ED mainly function as the flexor and the extensor of the middle phalanges of the four fingers, respectively. FDI and FDM, on the other hand, function as the flexor/abductor of the index finger and the flexor of the little finger, respectively. EMG signal was sampled at 5,000 Hz. First, for each muscle, bias and linear trends were removed from the raw EMG signal. Then, to calculate EMG power, full wave rectification was applied, followed by low-pass filtering (butterworth filter, f_co_ = 20 Hz) to obtain the EMG signal envelope. Also, RMS values were calculated on the detrended and high-pass filtered (butterworth filter, f_co_ = 10 Hz) EMG signal. For each extrinsic muscle and each block, EMG power and RMS were averaged across the whole block, as FDS and ED were activated throughout the task block (during all four finger movements). However, for intrinsic muscles, EMG power and RMS values were calculated and averaged over time intervals centered on the index and pinkie key presses (150 ms before and after each key press) in each block, as FDI and FDM are mainly activated during the index and pinkie movements, respectively. To correct for differences in the number of index (or pinkie) key presses across conditions, mean RMS values were divided by the square root of the number of key presses in each condition. Also, in order to obtain comparable measures across subjects, for each block, EMG power and RMS values were normalized by the average power and RMS values over all blocks of SS condition, respectively.

### Statistical Analyses

Results are shown as mean ± SEM. Based on previous studies of spinal cord and brain imaging, 25 subjects were deemed to yield sufficient power to detect changes within the sensorimotor network [54]. Data were checked for normality and equality of variance across conditions. Unless otherwise indicated, statistical significance was determined using repeated measures two-tailed *t*-tests (when comparing two conditions) or repeated measures ANOVAs (when comparing more than two conditions). Results were considered to be significant at *p* < 0.05.

## Supporting Information

S1 Data S1 Data provides the numerical values related to the graphs plotted in Figs 1D, 1E, 2 and 5B, S5 and S6 Figs. (XLSX)

S2 Data S2 Data contains the FSL Z-score images (Neuroimaging Informatics Technology Initiative [NIfTI-1] format) of the statistical maps reported in Figs 2–5. (ZIP)

S3 Data S3 Data contains the FSL Z-score images (NIfTI-1 format) of the statistical maps reported in S1, S2 and S4 Figs. (ZIP)

S1 Fig Neural correlates of motor practice in the brain. (A, B) Brain activation maps representing the main effect of practice during the complex sequence (CS; A) and simple sequence (SS; B) conditions are overlaid on the MNI template. Peaks of activity are located bilaterally in the primary sensorimotor cortex, right dorsal and ventral premotor cortices, supplementary motor area, anterior cingulate cortex, putamen, and cerebellar cortex, Lobules V and VI (corrected for family-wise error using GRF, Z > 2.3 and a cluster significance threshold of *p* < 0.01). Color-coded bars represent *Z* scores.
(TIF)

S2 Fig Individual level’s activation maps (*n* = 23) at the cervical level during motor sequence learning demonstrate a consistent cluster of activity around the C7 spinal level. Sagittal slices represent the main effect of practice during the CS condition (i.e., CS - baseline). In line with the results at the group level, the majority of subjects (21 out of 23) are showing clusters of activation between the C6 to C8 spinal segments. For illustration purposes, the activation map of each subject is registered to the anatomical space of a reference subject. Color-coded bars represent *Z* scores. The activity maps are thresholded at Z > 2.5, uncorrected.
(TIF)

S3 Fig The spinal cord mask centered on the C7 spinal level used in the ROI spinal cord analysis. Left panel shows the midsagittal view of the applied mask, spanning from the C6 to C8 spinal levels. Right panels show different spinal levels highlighted by the yellow straight lines on the left.
(TIF)

S4 Fig The group-level activation map showing the difference in modulation by performance speed between conditions. Left and right panels show the two significant activation clusters centered on the C7 and C8 spinal levels in the sagittal and axial views, respectively. The BOLD activities in these clusters show significantly greater modulation by performance speed in the CS condition compared to the SS condition. The color bars indicate *Z-*score values; activation maps are corrected for multiple comparisons using GRF, *p* < 0.01.
(TIF)

S5 Fig The motor learning curves as measured by speed performance in the control group. This graph shows performance speeds (i.e., block duration) averaged across all subjects who participated in the EMG recording control group (*n* = 10) during the different blocks of practice in the CS (red) and SS (blue) conditions. Error bars represent SEM.
(TIF)

S6 Fig Normalized EMG power and RMS were not significantly different between the CS and SS conditions in any of the recorded muscles. (A–D) show EMG power (left panels) and EMG RMS (right panels) averaged across all subjects during the different blocks of practice in the CS (red) and SS (blue) conditions. EMG activity was recorded from the (A) FDS: flexor digitorum superficialis, (B) ED: extensor digitorum, (C) FDI: first dorsal interosseous, and (D) FDM: flexor digiti minimi muscle. The condition by blocks interaction *p*-values are 0.22, 0.53, 0.76, and 0.89 in (A–D) left panels, and 0.2, 0.6, 0.55, and 0.68 in (A–D) right panels, respectively (two-factor within-subject repeated measures ANOVA, *df* = 11). The main effect of condition *p*-values are 0.15, 0.66, 0.22, and 0.14 in (A–D) left panels, and 0.25, 0.36, 0.44, and 0.20 in (A-D) right panels, respectively. Also, the main effect of blocks *p*-values are 0.18, 0.19, 0.07, and 0.36 in (A–D) left panels, and 0.15, 0.26, 0.57, and 0.07 in (A–D) right panels, respectively. EMG power and RMS values were normalized by their average values during SS condition blocks. Error bars represent SEM.
(TIF)

S7 Fig Example of a subject-specific mask used in the spinal cord analysis overlaid on the anatomical image of the reference subject. The mask was defined in the functional space of each subject and included at least two additional voxels outside the spinal cord along the anteroposterior and mediolateral axes. (A–C) show the mask on the sagittal, coronal, and axial views. The mask included cervical regions from the C2 vertebral level down to the T1 vertebral level (shown separately in panel C).
(TIF)

S8 Fig Preprocessing steps in functional spinal cord analysis, including unwarping, segmentation, and realignment to a reference subject. GRE field map unwarping was used to correct for geometrical distortions in both the brain and spinal cord (B0-field map correction). As shown here for a representative subject, this step allowed us to correct for the large spatial distortions around the subject’s neck at the level of the spinal cord. For each subject, a mask of the cervical cord was then manually generated and applied to the functional data (cervical cord segmented). Finally, the center of the cervical cord in each slice was manually marked and shifted on the lateral and anteroposterior axes to match that of a reference subject (realigned to reference).
(TIF)

S1 Table Seed regions used in the first model of the conditional spinal cord–brain activity analysis. Peaks of activity were extracted from the main effect of practice during both CS and SS conditions. For each peak, the anatomical label, MNI coordinates, and the associated *Z*-scores from the CS and SS conditions’ activation maps (S1A and S1B Fig, respectively) are reported. BA: Broadman area.
(DOCX)

S2 Table Seed regions used in the second model of the conditional spinal cord–brain activity analysis. Table reports activation peaks related to the significant brain clusters that were modulated by the performance speed during the CS condition only (Fig 3 cortical and subcortical levels). For each peak of activity, the anatomical label, MNI coordinates, the corrected cluster-level *p*-value, and the associated *Z*-score are reported.
(DOCX)

S3 Table Adjusted R^2^ values for different hierarchical regression models. Each row reports the mean adjusted R^2^ value of the regression models (averaged across subjects), which explain each individual’s time course of performance speed in the CS condition using different combinations of cortical, subcortical, and spinal cord components’ time series. On average, nine components per cluster were extracted for each subject using principal components analysis and an adaptive pruning algorithm [57]. Note that the spinal cord accounts for 0.021 / 0.086 = 24% of total variability explained by the full model (Cortical + Subcortical + Spinal cord), among which (0.086–0.069) / 0.021 = 81% is independent from the contribution of cortical and subcortical regions.
(DOCX)

## Abbreviations

BOLD: blood oxygenated level dependent
CB: cerebellum
CNS: central nervous system
CS: complex sequence
ED: extensor digitorum
EMG: electromyography
EPI: echo-planar imaging
FDI: first dorsal interosseous
FDM: flexor digiti minimi
FDS: flexor digitorum superficialis
fMRI: functional magnetic resonance imaging
GLM: general linear model
GRF: Gaussian random field theory
H-reflex: Hoffman reflex
HRF: hemodynamic response function
M1: primary motor cortex
MPRAGE: magnetization-prepared rapid gradient echo
MNI: Montreal Neurological Institute
MSL: motor sequence learning
PPI: psychophysiological interaction
RMS: root-mean-square
SNR: signal-to-noise ratio
ROI: region of interest
S1: primary somatosensory cortex
SEEP: signal enhancement by extravascular water protons
SEM: standard error of the mean
SMC: primary sensorimotor cortex
SS: simple sequence
T1: first thoracic
