# Supplementary figures and images for: Simultaneous Brain–Cervical Cord fMRI Reveals Intrinsic Spinal Cord Plasticity during Motor Sequence Learning
Source: PLoS Biol. 2015 Jun 30;13(6):e1002186. doi: 10.1371/journal.pbio.1002186 (PMC4488354; doi:10.1371/journal.pbio.1002186)

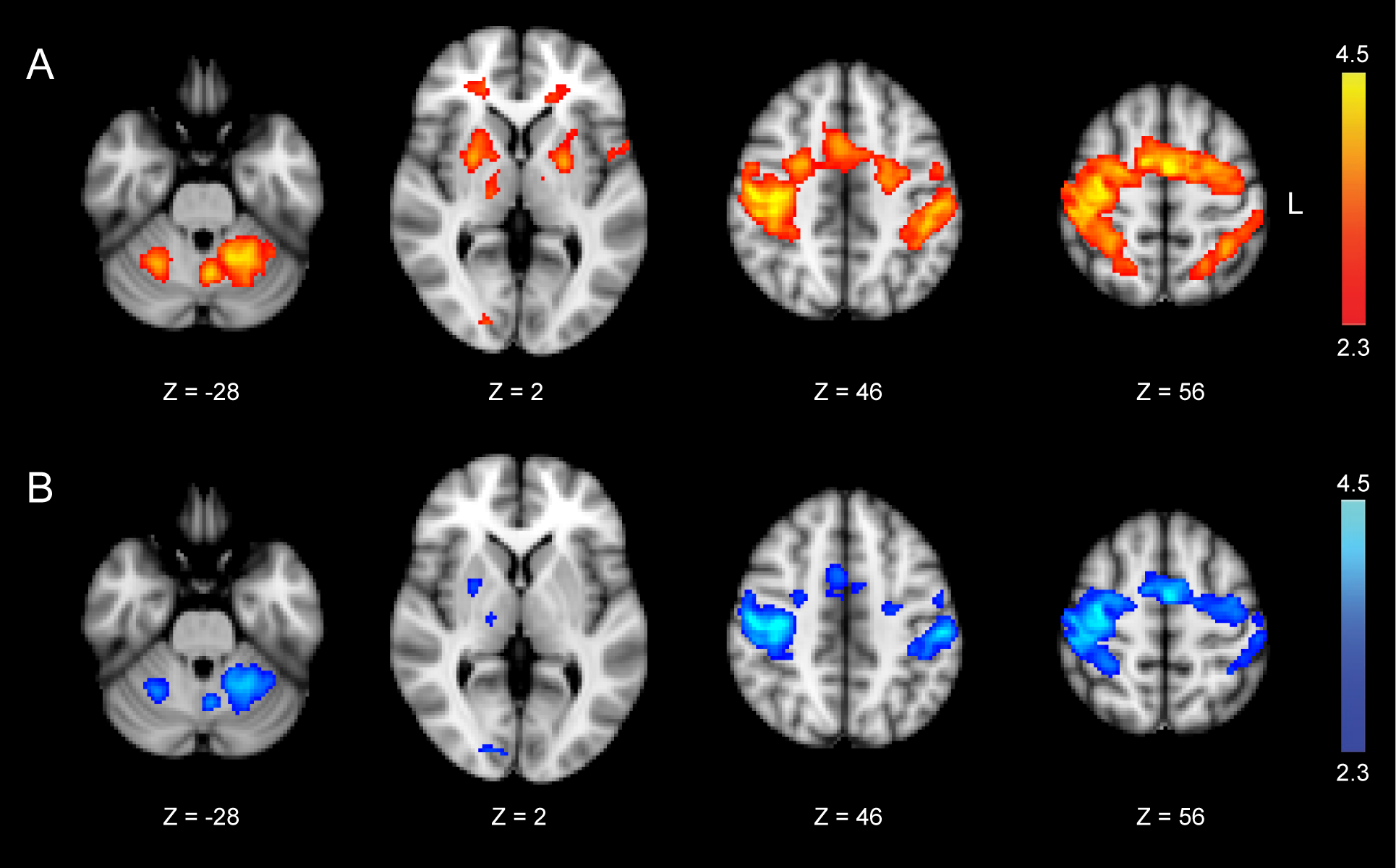

Supplement: S1 Fig — (A, B) Brain activation maps representing the main effect of practice during the complex sequence (CS; A) and simple sequence (SS; B) conditions are overlaid on the MNI template. Peaks of activity are located bilaterally in the primary sensorimotor cortex, right dorsal and ventral premotor cortices, supplementary motor area, anterior cingulate cortex, putamen, and cerebellar cortex, Lobules V and VI (corrected for family-wise error using GRF, Z > 2.3 and a cluster significance threshold of p < 0.01). Color-coded bars represent Z scores. (TIF) [file pbio.1002186.s004.tif]

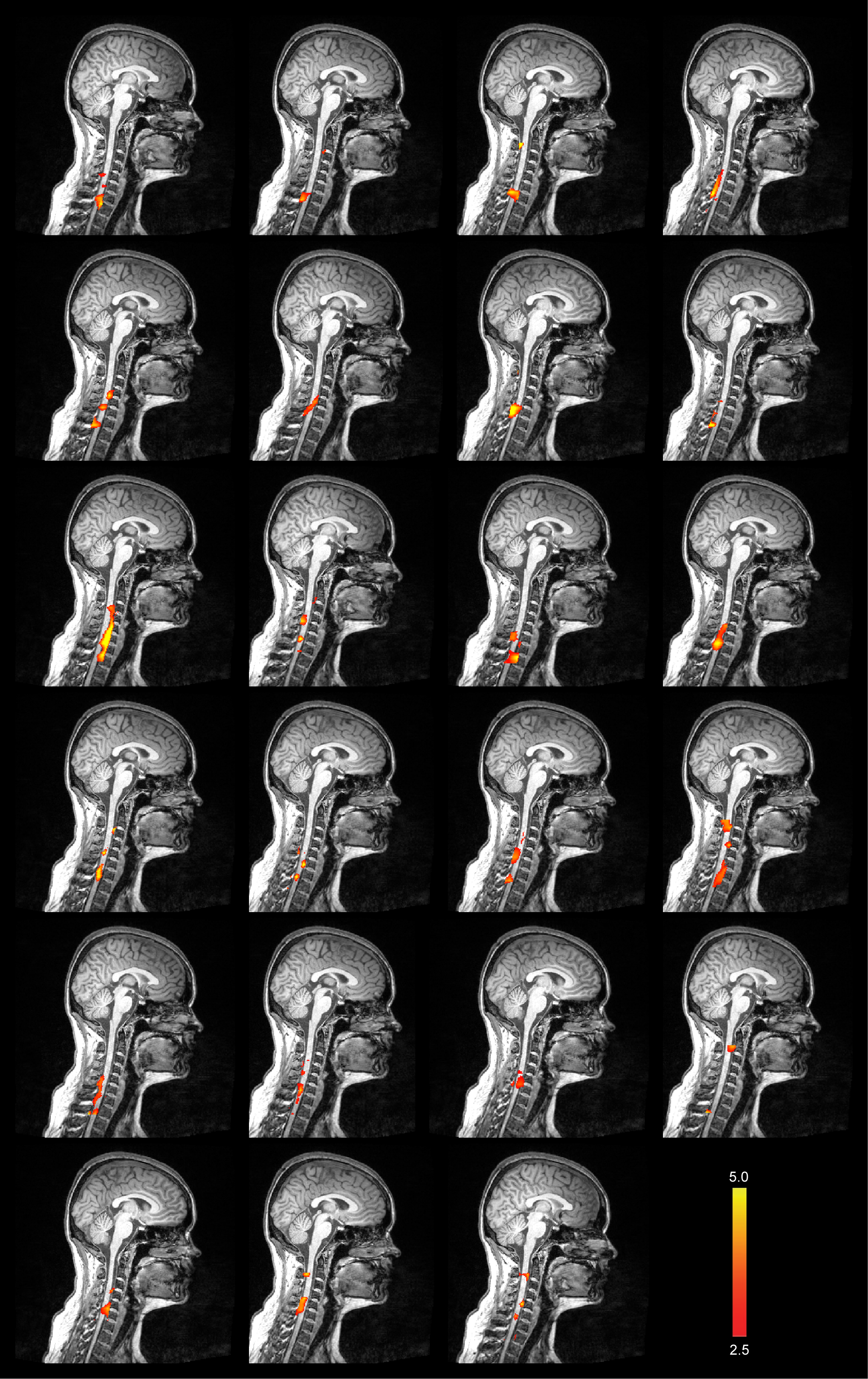

Supplement: S2 Fig — Sagittal slices represent the main effect of practice during the CS condition (i.e., CS - baseline). In line with the results at the group level, the majority of subjects (21 out of 23) are showing clusters of activation between the C6 to C8 spinal segments. For illustration purposes, the activation map of each subject is registered to the anatomical space of a reference subject. Color-coded bars represent Z scores. The activity maps are thresholded at Z > 2.5, uncorrected. (TIF) [file pbio.1002186.s005.tif]

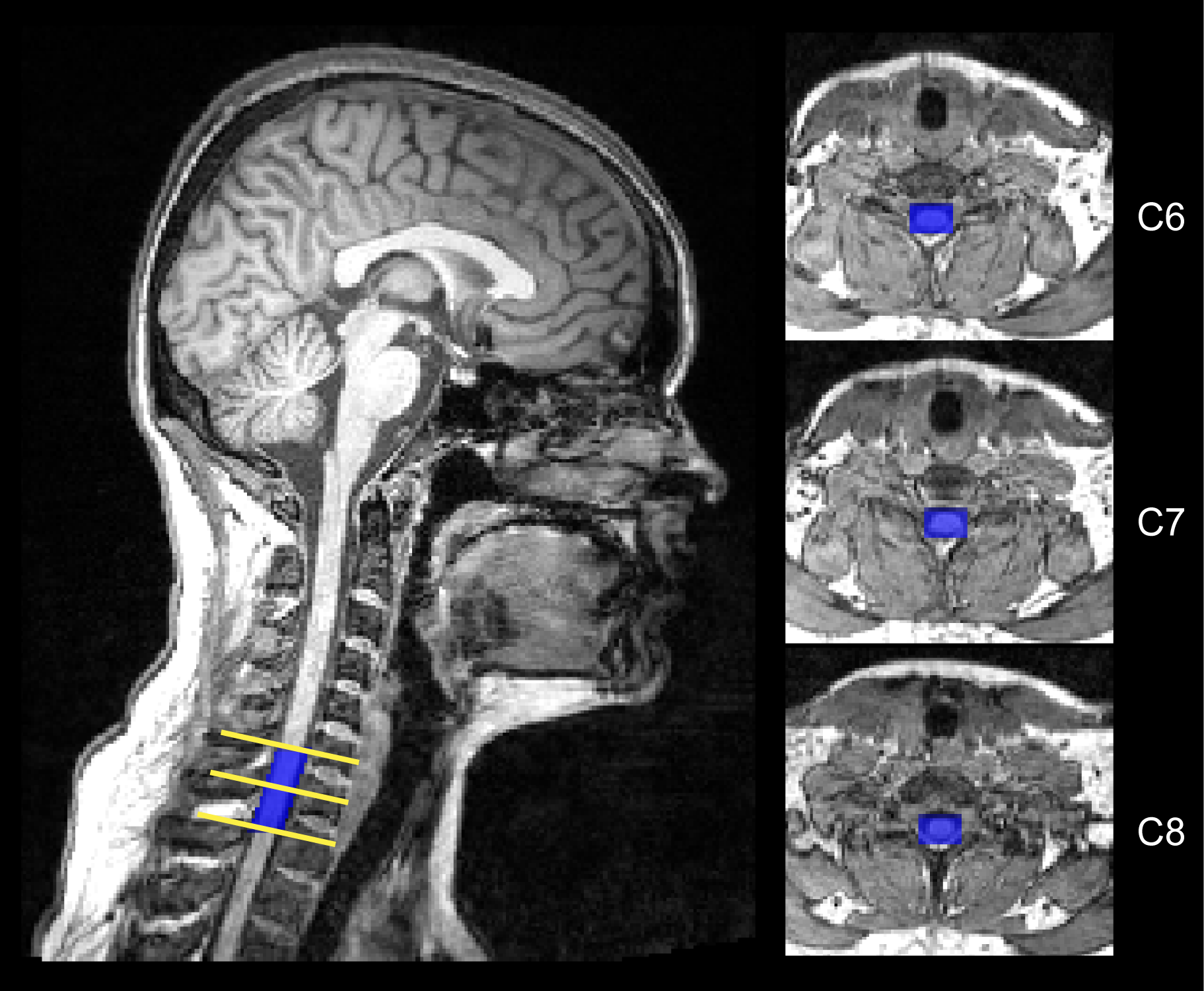

Supplement: S3 Fig — Left panel shows the midsagittal view of the applied mask, spanning from the C6 to C8 spinal levels. Right panels show different spinal levels highlighted by the yellow straight lines on the left. (TIF) [file pbio.1002186.s006.tif]

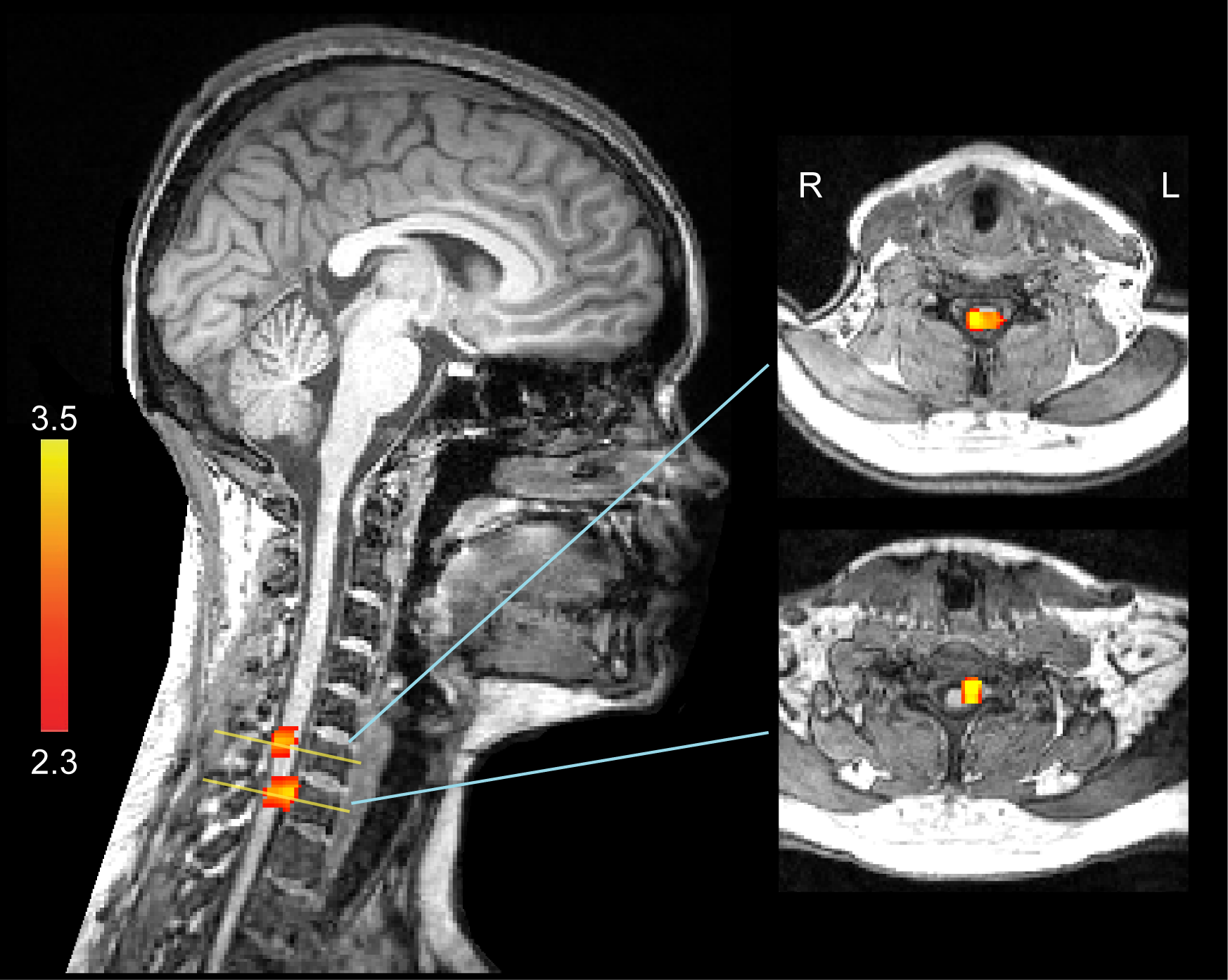

Supplement: S4 Fig — Left and right panels show the two significant activation clusters centered on the C7 and C8 spinal levels in the sagittal and axial views, respectively. The BOLD activities in these clusters show significantly greater modulation by performance speed in the CS condition compared to the SS condition. The color bars indicate Z-score values; activation maps are corrected for multiple comparisons using GRF, p < 0.01. (TIF) [file pbio.1002186.s007.tif]

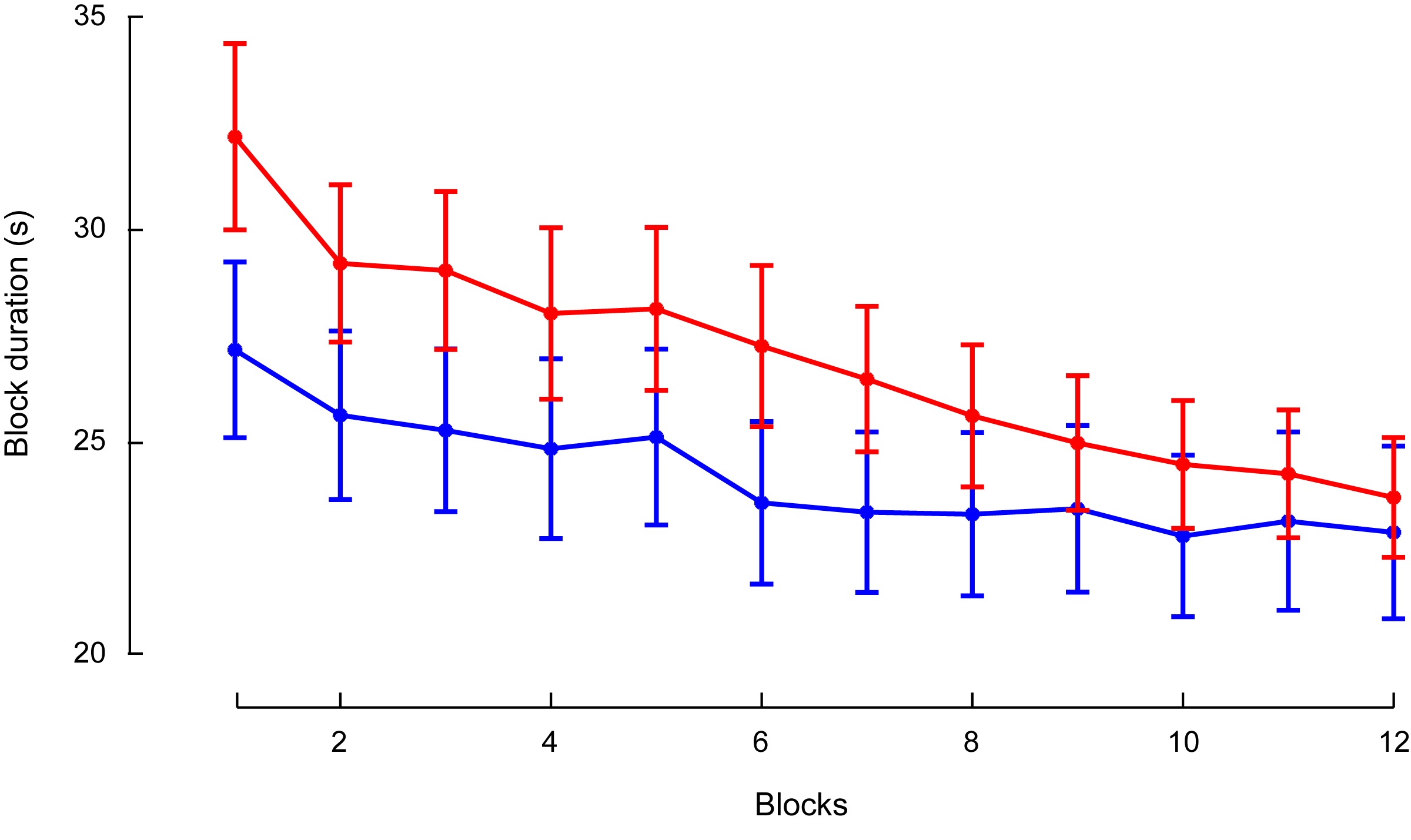

Supplement: S5 Fig — This graph shows performance speeds (i.e., block duration) averaged across all subjects who participated in the EMG recording control group (n = 10) during the different blocks of practice in the CS (red) and SS (blue) conditions. Error bars represent SEM. (TIF) [file pbio.1002186.s008.tif]

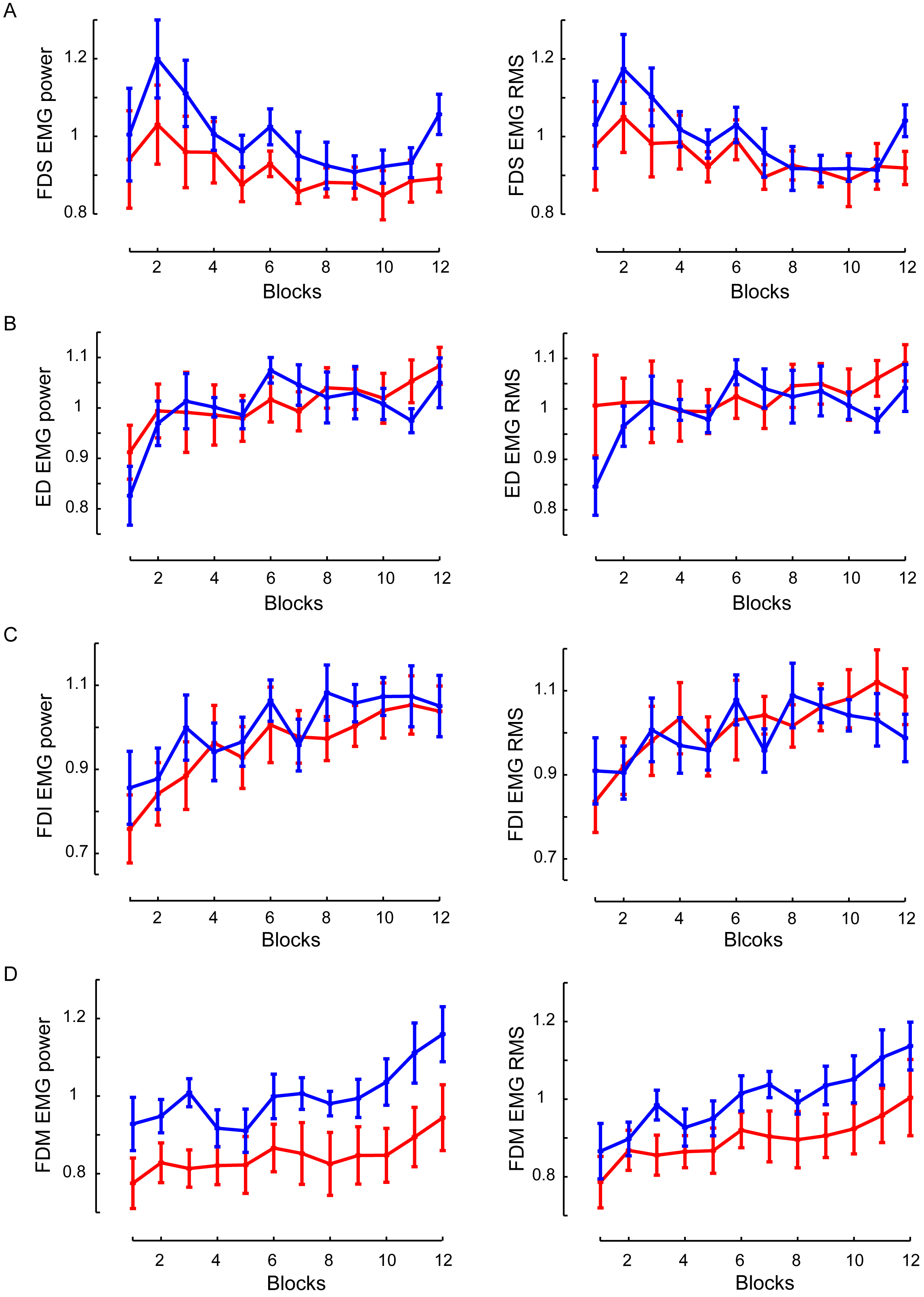

Supplement: S6 Fig — (A–D) show EMG power (left panels) and EMG RMS (right panels) averaged across all subjects during the different blocks of practice in the CS (red) and SS (blue) conditions. EMG activity was recorded from the (A) FDS: flexor digitorum superficialis, (B) ED: extensor digitorum, (C) FDI: first dorsal interosseous, and (D) FDM: flexor digiti minimi muscle. The condition by blocks interaction p-values are 0.22, 0.53, 0.76, and 0.89 in (A–D) left panels, and 0.2, 0.6, 0.55, and 0.68 in (A–D) right panels, respectively (two-factor within-subject repeated measures ANOVA, df = 11). The main effect of condition p-values are 0.15, 0.66, 0.22, and 0.14 in (A–D) left panels, and 0.25, 0.36, 0.44, and 0.20 in (A-D) right panels, respectively. Also, the main effect of blocks p-values are 0.18, 0.19, 0.07, and 0.36 in (A–D) left panels, and 0.15, 0.26, 0.57, and 0.07 in (A–D) right panels, respectively. EMG power and RMS values were normalized by their average values during SS condition blocks. Error bars represent SEM. (TIF) [file pbio.1002186.s009.tif]

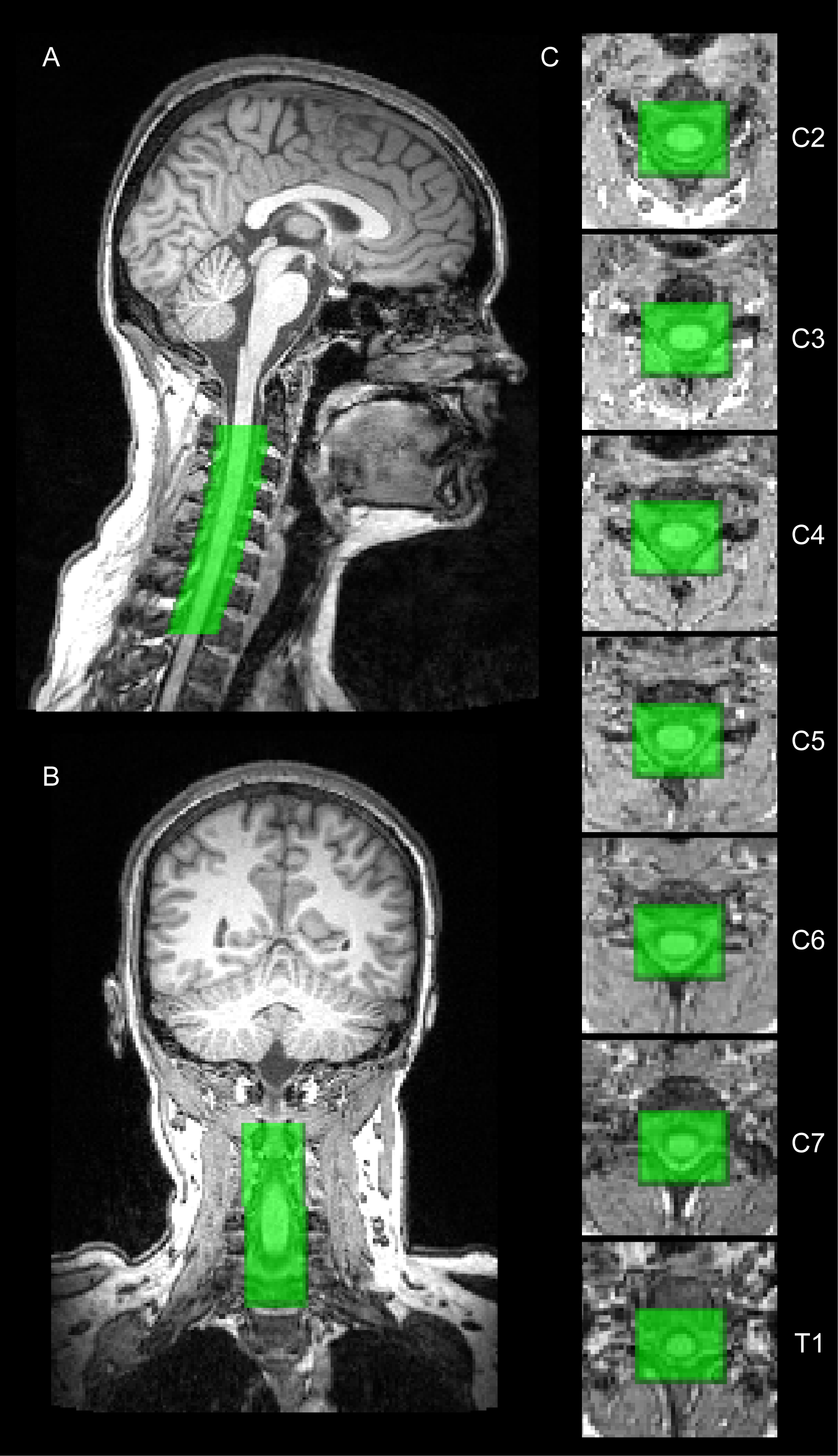

Supplement: S7 Fig — The mask was defined in the functional space of each subject and included at least two additional voxels outside the spinal cord along the anteroposterior and mediolateral axes. (A–C) show the mask on the sagittal, coronal, and axial views. The mask included cervical regions from the C2 vertebral level down to the T1 vertebral level (shown separately in panel C). (TIF) [file pbio.1002186.s010.tif]

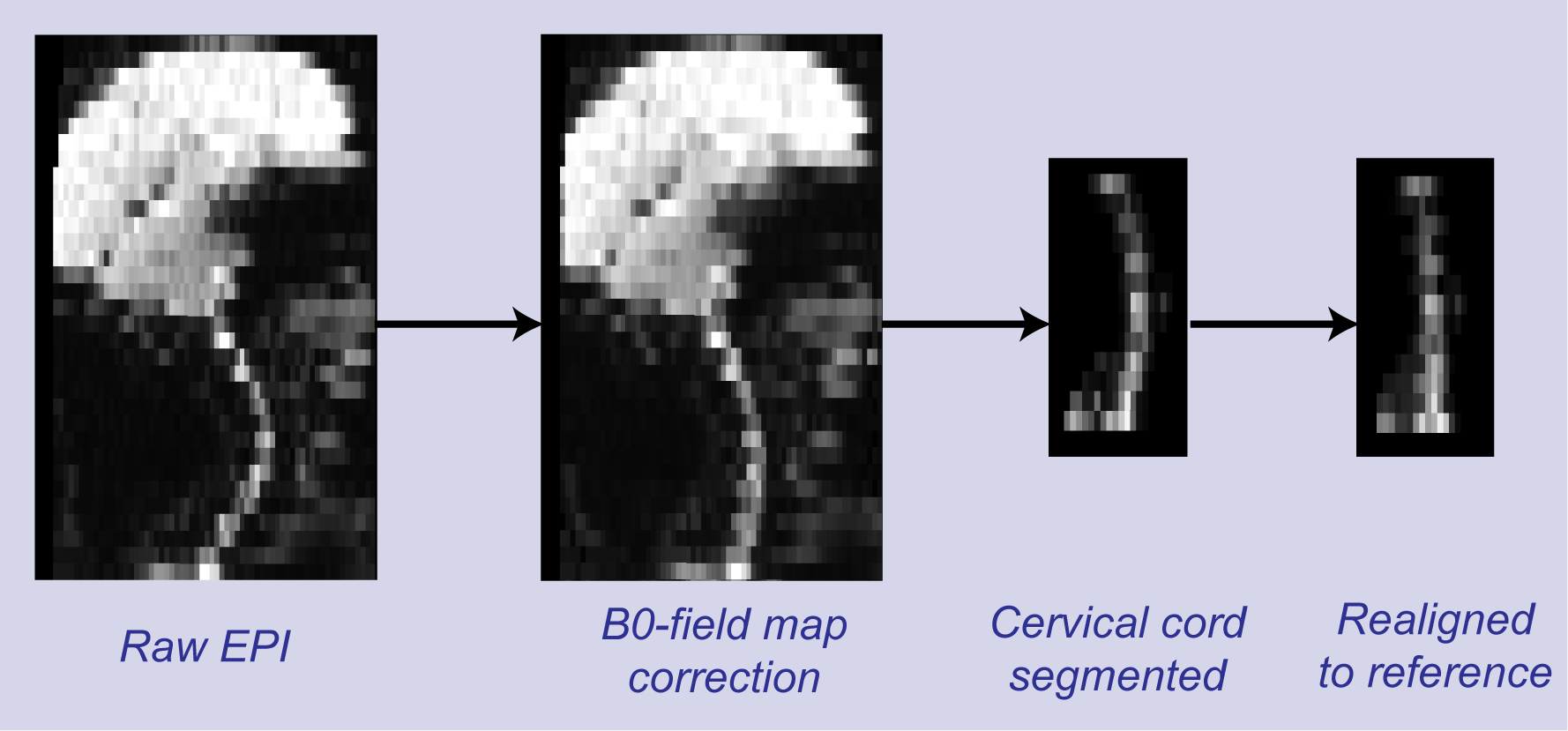

Supplement: S8 Fig — GRE field map unwarping was used to correct for geometrical distortions in both the brain and spinal cord (B0-field map correction). As shown here for a representative subject, this step allowed us to correct for the large spatial distortions around the subject’s neck at the level of the spinal cord. For each subject, a mask of the cervical cord was then manually generated and applied to the functional data (cervical cord segmented). Finally, the center of the cervical cord in each slice was manually marked and shifted on the lateral and anteroposterior axes to match that of a reference subject (realigned to reference). (TIF) [file pbio.1002186.s011.tif]
