# Supplementary material for: Simultaneous Brain–Cervical Cord fMRI Reveals Intrinsic Spinal Cord Plasticity during Motor Sequence Learning
Source: PLoS Biol. 2015 Jun 30;13(6):e1002186. doi: 10.1371/journal.pbio.1002186 (PMC4488354; doi:10.1371/journal.pbio.1002186)
Supplement: S1 Table — Peaks of activity were extracted from the main effect of practice during both CS and SS conditions. For each peak, the anatomical label, MNI coordinates, and the associated Z-scores from the CS and SS conditions’ activation maps (S1A and S1B Fig, respectively) are reported. BA: Broadman area. (DOCX) [file pbio.1002186.s012.docx]

**S1 Table.**

| **Anatomical label** | **MNI coordinates** | | | **Peak’s *Z*-value** | | | |  |
| --- | --- | --- | --- | --- | --- | --- | --- | --- |
|  | ***x y z*** | | | **SS** | | **CS** | |  |
| Right primary motor cortex, BA4 | 38 | -20 | 50 | | 6.8 | | 6.9 | |
| Right dorsal premotor cortex, BA6 | 36 | -10 | 58 | | 6.1 | | 6.0 | |
| Right ventral premotor cortex, BA6 | 28 | -6 | 48 | | 4.5 | | 3.9 | |
| Supplementary motor area (SMA) | -2 | -4 | 56 | | 5.9 | | 5.8 | |
| Right anterior cingulate cortex | 4 | 8 | 42 | | 4.5 | | 4.6 | |
| Right primary somatosensory cortex, BA3 | 42 | -28 | 60 | | 6.0 | | 5.5 | |
| Left Cerebellum, lobule VI | -24 | -54 | -28 | | 5.4 | | 5.0 | |
| Left primary motor cortex, BA4 | -38 | -16 | 56 | | 4.8 | | 4.3 | |
