# Supplementary material for: Simultaneous Brain–Cervical Cord fMRI Reveals Intrinsic Spinal Cord Plasticity during Motor Sequence Learning
Source: PLoS Biol. 2015 Jun 30;13(6):e1002186. doi: 10.1371/journal.pbio.1002186 (PMC4488354; doi:10.1371/journal.pbio.1002186)
Supplement: S2 Table — Table reports activation peaks related to the significant brain clusters that were modulated by the performance speed during the CS condition only (Fig 3 cortical and subcortical levels). For each peak of activity, the anatomical label, MNI coordinates, the corrected cluster-level p-value, and the associated Z-score are reported. (DOCX) [file pbio.1002186.s013.docx]

**S2 Table.**

| **Anatomical label** | ***P* _cluster_** | ***Z* value** | **MNI coordinate** | | |
| --- | --- | --- | --- | --- | --- |
|  |  |  | ***x*** | ***y*** | ***z*** |
| Right primary motor cortex, BA4 | 0.0002 | 3.7 | 36 | -24 | 50 |
| Right primary somatosensory cortex, BA1/2 |  | 3.4 | 46 | -20 | 62 |
| Right dorsal premotor cortex, BA6 |  | 3.1 | 30 | -16 | 70 |
| Left cerebellum, Lobule VI | 0.0001 | 3.6 | -20 | -64 | -20 |
| Left cerebellum, Lobule V |  | 3.2 | -16 | -46 | -24 |
| Right putamen | 0.02 | 3.4 | 28 | 4 | -8 |
