# Supplementary material for: Simultaneous Brain–Cervical Cord fMRI Reveals Intrinsic Spinal Cord Plasticity during Motor Sequence Learning
Source: PLoS Biol. 2015 Jun 30;13(6):e1002186. doi: 10.1371/journal.pbio.1002186 (PMC4488354; doi:10.1371/journal.pbio.1002186)
Supplement: S3 Table — Each row reports the mean adjusted R2 value of the regression models (averaged across subjects), which explain each individual’s time course of performance speed in the CS condition using different combinations of cortical, subcortical, and spinal cord components’ time series. On average, nine components per cluster were extracted for each subject using principal components analysis and an adaptive pruning algorithm [57]. Note that the spinal cord accounts for 0.021 / 0.086 = 24% of total variability explained by the full model (Cortical + Subcortical + Spinal cord), among which (0.086–0.069) / 0.021 = 81% is independent from the contribution of cortical and subcortical regions. (DOCX) [file pbio.1002186.s014.docx]

**S3 Table.**

| **Regression model** | $\mathbf{Adjusted}\mathbf{R}^{\mathbf{2}}$ |
| --- | --- |
| Cortical | 0.054 |
| Subcortical | 0.0265 |
| Spinal cord | 0.021 |
| Cortical + Subcortical | 0.069 |
| Cortical + Spinal cord | 0.072 |
| Subcortical + Spinal cord | 0.047 |
| Cortical + Subcortical + Spinal cord | 0.086 |
